# Uncertainty in near-term global surface warming linked to tropical Pacific climate variability

**DOI:** 10.1038/s41467-019-09761-2

**Published:** 2019-04-30

**Authors:** Mohammad Hadi Bordbar, Matthew H. England, Alex Sen Gupta, Agus Santoso, Andréa S. Taschetto, Thomas Martin, Wonsun Park, Mojib Latif

**Affiliations:** 1 GEOMAR Helmholtz Centre for Ocean Research Kiel, 24105 Kiel, Germany; 2 Climate Change Research Centre and ARC Centre of Excellence for Climate Extremes, University of New South Wales, New South Wales, 2052 Australia; 3 Centre for Southern Hemisphere Oceans Research (CSHOR), CSIRO Oceans and Atmosphere, Hobart, 7004 Australia; 4 Excellence Cluster “The Future Ocean”, University of Kiel, 24098 Kiel, Germany

**Keywords:** Atmospheric science, Climate change, Ocean sciences

## Abstract

Climate models generally simulate a long-term slowdown of the Pacific Walker Circulation in a warming world. However, despite increasing greenhouse forcing, there was an unprecedented intensification of the Pacific Trade Winds during 1992–2011, that co-occurred with a temporary slowdown in global surface warming. Using ensemble simulations from three different climate models starting from different initial conditions, we find a large spread in projected 20-year globally averaged surface air temperature trends that can be linked to differences in Pacific climate variability. This implies diminished predictive skill for global surface air temperature trends over decadal timescales, to a large extent due to intrinsic Pacific Ocean variability. We show, however, that this uncertainty can be considerably reduced when the initial oceanic state is known and well represented in the model. In this case, the spatial patterns of 20-year surface air temperature trends depend largely on the initial state of the Pacific Ocean.

## Abstract (web summary)

Large uncertainty exists in projecting future 20-year global warming trends due to intrinsic tropical Pacific climate variability. Here the authors show that knowledge of the state of the Pacific Ocean can significantly reduce this uncertainty via the use of initialized decadal climate forecasts.

## Introduction

The tropical Pacific covers a vast area, ~10%, of the Earth’s surface and is subject to intense ocean-atmosphere exchanges of momentum, heat, and moisture, thus playing a vital role in modulating regional and global climate^1–3^. This region has undergone significant ocean and atmospheric changes^4^ over two recent decades (1992–2011). During the latter half of this period and despite the steady buildup of atmospheric greenhouse gases, the rate of global surface warming slowed, at the same time that there were persistently cold surface and subsurface temperature anomalies in the central and eastern tropical Pacific Ocean^4–6^. This multi-decadal change is La Niña-like, in that the period also saw anomalously wet conditions and sea-level rise in the western Pacific, a cool and dry eastern Pacific, an intensified Indonesian Throughflow and an increase in wind-driven Ekman divergence away from the equator^4,6,7^.

These changes were associated with an unprecedented intensification of the equatorial trade winds that form the surface component of the Pacific Walker Circulation (PWC)^4–6,8^. More broadly, Pacific trade wind variations are associated with sea surface temperature (SST) anomalies, predominantly in the Niño 3.4 region (5°N–5°S, 170°W–120°W), basin-wide changes in sea-level pressure (SLP) and changes in upper-ocean thermal structure^4,6,9^. Therefore, PWC anomalies are an indicator of the state of the tropical Pacific surface climate state^4,6,8,9^.

Large internal decadal variability is evident in the tropical Pacific in both observations and climate simulations, which can enhance or reduce the rate of globally averaged surface warming^6,10–15^. On multi-decadal timescales, an ENSO-like pattern in anomalous SST, known as the Interdecadal Pacific Oscillation (IPO), is observed and simulated in climate models^9,16–22^. Changes in the IPO phase have been shown to coincide with significant changes in the tropical and subtropical Pacific climate and globally averaged surface air temperature (SAT)^4,6,23,24^ (hereafter, termed the global mean temperature; GMT). For example, the anomalous decadal intensification of the Pacific trade winds between 1992 and 2011 was associated with a decadal shift from the warm to cold phase of the IPO during the late 1990 s or early 2000s^4^. Thus, understanding the causes of transitions in the IPO and improving its simulation in climate models is vital to enhancing our ability to make skillful decadal climate predictions^1,12,25^. At present, perhaps owing to the large impact of stochastic atmospheric variability on IPO evolution, decadal predictions of the IPO exhibit limited skill; compared, for example, with the Atlantic Multidecadal Oscillation^21,26^.

The sign and magnitude of Pacific trade winds response to increased atmospheric greenhouse gas concentrations (GHGs) is still uncertain. The majority of models participating in Coupled Model Intercomparison Project Phase 5 project a future decrease in the strength of the Pacific trade^4,27–29^, although there is large inter-model spread in the magnitude of the change^30,31^. It is consistent with theoretical arguments relating tropical warming to a slowdown of the Walker Circulation^3,8^. Yet as noted, during 1992–2011 there was an unprecedented increase in observed trade wind strength; with no model from the Coupled Model Intercomparison Project Phase 5 able to simulate the magnitude of this intensification^4,24^. The magnitude of the Pacific trade wind acceleration has been linked with internal variability^6,20,29^, as well as multi-decadal surface warming in the Atlantic^32^ and Indian^33^ Oceans. Other work also indicates that low-frequency variability is generally underestimated in the current generation of climate models^6,20,29^.

The role of tropical Pacific internal variability in the recent global surface warming slowdown is still unclear. Here, we investigate the influence of low-frequency changes in the Pacific climate in modulating GMT. Using multiple large ensembles of global warming simulations subject to identical external forcing, but starting from different initial conditions (Methods; Supplementary Table 1), we explore the impacts of internal variability on tropical Pacific climate hindcasts and projections and its relationship to GMT. We mainly focus on 20-year timescale, which is beyond the target range of decadal prediction but not so long that multi-decadal variability is dominated by anthropogenic GHG forcing^34^.

In this study, we mainly focus on the fluctuations of Pacific trade winds, among other elements of tropical Pacific climate variability. The wind forcing is an integral element of upper-ocean circulation, fundamentally altering the state of the ocean, both in the equatorial and off-equatorial regions. In particular, the surface wind stress field drives the redistribution of ocean heat content via the shallow meridional overturning cells and equatorial thermocline displacements. Wind forcing also alters the tropical Ekman transport that regulates ocean heat and water mass exchange between the tropics and subtropics over decadal timescales^4,6,7^.

## Results

### Impact of tropical Pacific climate variability on GMT

**Fig. 1:**
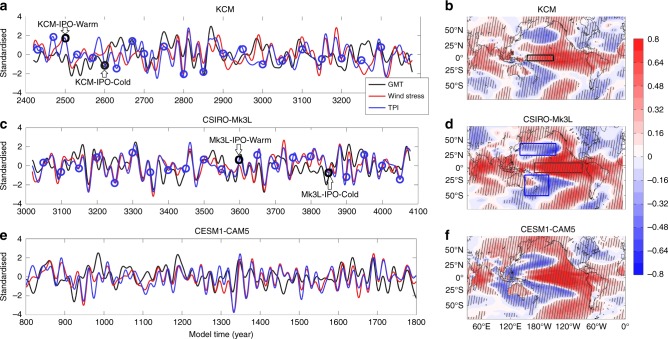
Time series of tropical Pacific zonal wind stress, globally averaged SAT, and the Tripole Index in the three climate models. Time series of low-frequency fluctuations (11-yr low passed filter) in (**a**, **c**, **e**) zonal wind stress over western equatorial Pacific (red solid lines), globally averaged SAT (black solid lines), and the Tripole Index (TPI; blue solid lines) from **a** KCM, **c** Mk3L, and **e** CESM-CAM5 control runs (all time series have been standardized). Blue (black) circles in **a** and **c** denote the initial year of the KCM-ICs (the KCM-IPO-Cold and the KCM-IPO-Warm) and the Mk3L-ICs (the Mk3L-IPO-Cold and the Mk3L-IPO-Warm) global warming simulations, respectively. **b**, **d**, **f** panels display the corresponding correlation between low-frequency fluctuations in the equatorial zonal wind stress (11-yr low passed filter) and local SAT anomalies computed from the control runs. Boxes on **d** indicate locations used for the calculation of the TPI. Hatching in **b**, **d**, **f** show where the correlation is statistically significant at 99% confidence level (accounting for autocorrelation in the time series)

**Fig. 2:**
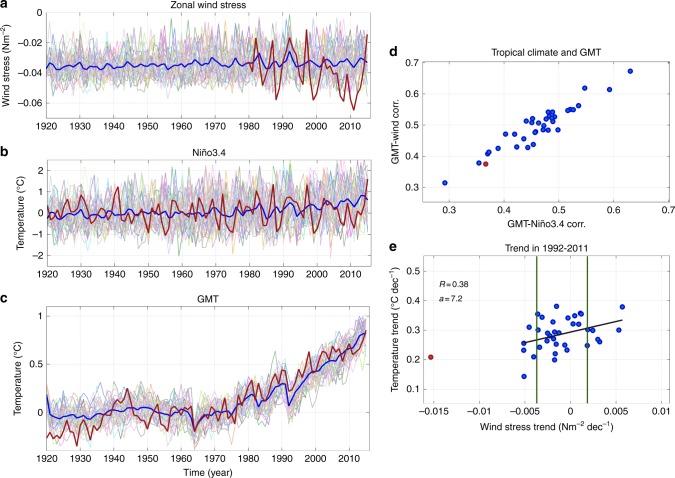
Observed and simulated Pacific trade winds, Niño3.4 index and global mean temperature. Time series of **a** annual mean equatorial zonal wind stress (areal average over the rectangular box defined in Fig. 1b; Nm^−2^), **b** areal averaged sea surface temperature over Niño3.4 region (170°W–120°W, 5°S–5°N; Niño3.4 index; °C) and **c** globally averaged SAT anomaly (°C) from individual members of the CESM-Hind-Proj global warming experiments (thin colored lines). Time series in **a** and **b** are computed relative to 1951–1980 baseline. The associated ensemble means and observations are shown as thick blue and red lines, respectively. Owing to large observational uncertainty in pre-satellite era^6^, observed wind stress is only shown after 1979. Shown in **d** is the annual correlation coefficient between the GMT and Niño3.4 index (horizontal axis) and between the GMT and the zonal wind stress (vertical axis). **e** 20-year trends in the zonal wind stress (Nm^−2^ /decade) and the GMT (°C/decade) for 1992–2011. In **d** and **e**, blue (red) circles denote the individual ensemble members (observation). The vertical solid lines in **e** indicate the mean ± Std of the trend in the wind stress and black solid lines denote the regression lines between GMT and wind stress trends. Also shown near the top-left corner of **e** are the correlation between the wind stress and GMT trends (*R*) and the slope of the regression line (*a*). Please note that in **d**, the linear trend was subtracted from all data sets prior to computing the correlation

Figure 1a–e show normalized time series of the low-frequency fluctuation in the tropical Pacific zonal wind stress, the Tripole Index^35^ (TPI, Methods), a proxy for the IPO, and the GMT obtained from the unforced control runs. The linear correlation between the annual mean wind stress and Niño3.4 index exceeds 0.86 in all three control runs (Supplementary Table 2). This indicates that the wind stress and Niño3.4 are strongly coupled, and thus the results of this study are qualitatively the same if the Niño 3.4 index, instead of Pacific trade winds, is considered (see also Fig. 2d). In general, acceleration (weakening) of the equatorial trade winds are associated with a decreased (increased) GMT with correlations of 0.31, 0.56 and 0.42 in the KCM, the CSIRO-Mk3L and the CESM1-CAM5 control runs, respectively (all significant at the 99% level; Supplementary Table 2; see Supplementary Fig. 1). However, all models simulate certain periods when this wind-GMT relationship breaks down. This may be related to periods of only moderate trade wind variations, insufficient to lead to a sustained acceleration or deceleration of heat uptake in the subtropical overturning cells^4^. It could also be owing to regional climate variability originating in other ocean basins^29,34^.

To identify the regional impacts of the Pacific trade wind trends, we compute the correlation between low-frequency Pacific trade wind variations and local SAT anomalies in the control runs (Fig. 1b–f). The spatial structures exhibit similar patterns in all models. Weakened trade winds, i.e., eastward anomalies, are generally associated with warming in the central and eastern tropical Pacific, tropical and subtropical South America, and the western part of the Indian Ocean, whereas it is accompanied by cooling over the Northwest and Southwest Pacific and the eastern sector of North America. Over the Pacific basin, the pattern is somewhat symmetrical about the equator and reminiscent of the IPO^1,35^. Eastward decadal anomalies in the Pacific trade winds are associated with a weakening of the equatorial Ekman divergence over the equatorial Pacific, which reduces poleward surface heat transport. Changes in the trade winds can also affect the Pacific mid-latitudes via atmospheric teleconnections forced by equatorial Pacific SST anomalies^1^. Furthermore, the correlation in the North and South Pacific is large and of the same sign, indicating that decadal SAT anomalies in these regions evolve coherently to some extent. This is suggestive of a pacemaker role for the tropical Pacific in terms of broader scale climate variability over decadal and multi-decadal timescales^36^ (see also Supplementary Fig. 2). However, the details of the spatial patterns vary across the models (Fig. 1), which is not surprising given the different space-time resolutions, model physics (e.g., atmospheric convection, ocean mixing) and numerical schemes employed in the models.

These internal fluctuations can result in large multi-decadal GMT trends (Supplementary Fig. 3; see methods), the magnitude of which is model dependent. For example, 10, 20, and 30-year unforced trends on average can reach up to ±0.34, ±0.14, and ± 0.08 (°C/decade), respectively (Supplementary Fig. 3), which can exceed expected externally forced trends on shorter timescales.

### Uncertainty in near-term tropical Pacific climate hindcasts

We begin by estimating the uncertainty owing to internal variability in the 20-year climate hindcast by analyzing a large ensemble of simulations (CESM-Hind-Proj; 35 members) for the CESM1-CAM5 subject to historical radiative forcing (Methods). Owing to large uncertainty in pre-satellite data^6^, we only consider the observed wind stress after 1979. The time series of the simulated annual mean trade winds (Fig. 2a) exhibits a large spread across the ensemble members over the entire period, resulting in a wide range of plausible trajectories in the tropical Pacific climate. Despite this large spread, the time series of observed trade winds (shown after 1979 in Fig. 2a) exceeds the envelope simulated by the CESM1-CAM5 ensemble members (particularly in 2011). Indeed, the observed 1992–2011 trend is clearly exceptional compared to the corresponding trends from the model (Fig. 2e). This discrepancy is common to other climate models^4,24,29^ and could have a number of possible reasons. In particular, the magnitude of low-frequency variability may be systematically underestimated in models^4,6,29^. Also, the recent wind intensification may have been partly enhanced by changes in radiative forcing^6,24,32^, and these may not be properly simulated by the model^37,38^. However, the time series of observed SST over Niño3.4 region (Fig. 2b), which is another proxy for tropical Pacific climate variability, is well within the range spanned by the model. In each ensemble member, the annual anomaly correlation coefficient between the wind stress and the Niño 3.4 index is statistically significant (99% confidence level) and higher than 0.89.

With respect to the globally averaged SAT (GMT; Fig. 2c), whereas the ensemble-mean captures its overall evolution, the spread across individual members is notable. The ensemble member trends can vary by ~±0.4 °C per 96-years (1920–2015) relative to the multi-model mean trend; a significant fraction of the ensemble-mean warming trend of ~0.9 °C over the same 96-year period. The uncertainty in the forced component of the trend becomes larger at shorter timescales. For instance, the 20-year trend over 1992–2011 in the simulated GMT varies from 0.14 to 0.38 °C/decade across the ensemble set (Fig. 2e, Supplementary Table 3) whereas the ensemble mean is ~0.26 °C/decade. This range is approximately equal to the range of internally driven 20-year trends in the corresponding control run (Supplementary Fig. 3). The observed warming trend (0.2 °C/decade) lies within the simulated range (Fig. 2e), although close to the lowest end of the ensemble set.

For each ensemble member, the annual correlation between GMT and wind stress is statistically significant (at 99% confidence level) and varies from 0.32 to 0.66. For each ensemble member, a similar correlation is found between the GMT and Niño3.4 index (Fig. 2d). This implies using either the wind stress-based or SST-based indices (Niño3.4) yields very similar results for the tropical Pacific climate variability and its connection to GMT. Further, there is a clear relationship between the trend in GMT and trend in Pacific trade winds across the members from 1992 to 2011 (*r* = 0.38, statistically significant at the 99% confidence level): ensemble members with stronger easterly (westerly) trade wind trend anomalies show reduced (enhanced) rates of global surface warming (Fig. 2e). Although the 20-year GMT trends always remain positive, the 20-year weakening and strengthening of the Pacific trade winds are about equally likely (Fig. 2e). Although strengthening of the trade winds is capable of temporarily slowing global surface warming, it is not sufficient to reverse the positive GMT trend, particularly if GHG concentrations are increasing rapidly^39^. Even during the recent observed two-decade period of unprecedented intensified Pacific trade winds (1992–2011), the GMT trend remained positive^4^ (Fig. 2e). The component of the trend that is related to internal variability is reduced when longer timescales are considered (Supplementary Fig. 3) and any forced signal becomes more dominant. For example, the range of GMT 30-year trends is nearly half of that related to 20-year trend (Supplementary Fig. 3). At longer timescales, we expect less ensemble spread and more predictable forced trend, as the forcing signal increases in magnitude relative to the size of internal variability.

**Fig. 3:**
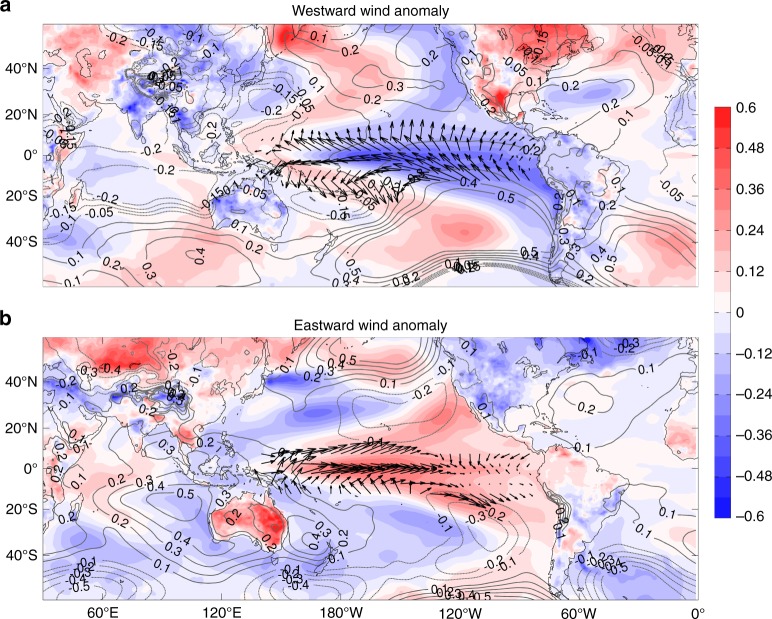
Composites of 20-year trends in SAT, SLP, and Pacific trade winds in the ensemble of climate hindcasts. Composites of 1992–2011 trends in SAT (shading; °C/decade), SLP (contours; hPa/decade) and wind stress (arrows; Nm^−2^/decade) from members from the CESM-Hind-Proj with large ( >1 s.d.) associated strengthening (**a**) or weakening (**b**) of the trade winds over the tropical Pacific (see Fig. 2e). The corresponding ensemble mean SAT trend (1992–2011), our estimate of the forced trend, is subtracted at each grid point. Note that the wind vectors are only shown for tropical Pacific sector

To understand how the climate state differs in relation to intensifying/weakening equatorial Pacific winds, we composite 20-year periods when these wind trend anomalies exceed ± 1 standard deviation (selected based on Fig. 2e). In the ensemble members with large westward 20-year trend in Pacific trade winds there is a cooling over the central and eastern tropical Pacific, the Australian continent, over East and Southeast Asia and the tropical and subtropical sector of South America along with warming over the Northwest and Southwest Pacific, and the eastern sector of North America (Fig. 3a). These changes are associated with enhanced (reduced) SLP in the eastern (western) Pacific. The spatial patterns are consistent with a strengthening of the PWC, increased equatorial upwelling and wind-driven Ekman divergence away from the equator, which cause anomalously large ocean heat uptake in the Pacific interior^4^. In fact these spatial patterns are similar to the observed trends over the 1992–2011 period (Supplementary Fig. 4), and are reminiscent of the negative phase of the IPO^4^. On the other hand, largely mirrored changes in SAT and SLP are simulated in the ensemble members associated with 20-year weakening trend in Pacific trade winds (Fig. 3b). This model analysis demonstrates the large and sometimes contrasting impact that internal decadal variability can have on 20-year trends. It also indicates the key role of large-scale ocean-atmosphere interactions in controlling the Pacific climate in the analyzed model. However, we cannot rule out remote influences from the Indian and Atlantic Oceans^32,33,40^. Moreover, internal variability adds large uncertainty in regional climate projections and can mask anthropogenic signals over the 20-year timescale^4,6,41–43^. For example, in most places the ensemble spread in 20-year trends in SLP (Supplementary Fig. 5b) exceeds the ensemble-mean trend (Supplementary Fig. 5a). In addition, the ratio of ensemble-mean trend to the ensemble spread, which defines the signal-to-noise ratio as a measure of the robustness of the forced trend, is in general smaller than 1.0 over the tropical region (Supplementary Fig. 5c), indicating the dominant impact of internal variability at this timescale^44^.

### Uncertainty in near-term tropical Pacific projections

**Fig. 4:**
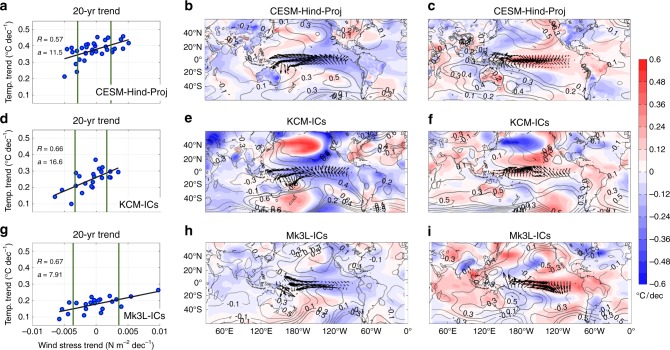
Composites of 20-year trends in SAT, SLP, and Pacific trade winds in three ensembles of a global warming simulation. **a**, **d**, **g** Scatter plot of 20-year trends in equatorial Pacific zonal wind stress (Nm^−2^/decade) and globally averaged SAT (°C/decade) from individual ensemble members (left panels). The vertical solid lines indicate the mean ± Std of the trend in the wind stress and black solid lines denote the regression lines between GMT and wind stress trends. The correlation between the wind stress and GMT trends and the slope of the regression lines are shown with *R* and *a* in the left panels. Other panels show composites of 20-year trends in SAT (shading; °C/decade), SLP (contours; hPa/decade), and wind stress (arrows; Nm^−2^/decade) based on ensemble members with wind stress trends less than (**b**, **e**, **h**) or exceeding (**c**, **f**, **i**) 1Std from CESM1-Hind-Proj (**a**–**c**) the KCM-ICs (**d**–**f**), and the Mk3L-ICs (**g**–**i**) ensemble of global warming simulations. **a**–**c** The trend is computed for the period corresponding to 2016–2035, whereas in **d**–**i** it is related to the first 20-year of the realizations. The corresponding ensemble mean SAT trend is subtracted from each grid point in **b**–**c**, **e**–**f**, **h**–**i**

Uncertainty in projected (CESM-Hind-Proj) and idealized transient (KCM-ICs, and Mk3L-ICs ensembles; starting from different oceanic and atmospheric Initial Conditions; Methods) SAT is next examined using large ensembles from three models. As with the historical ensemble (Fig. 2e) there is a large spread in 20-year trends in the Pacific trade winds and GMT across the ensemble members (Fig. 4a–g; Supplementary Table 3). Composites of 20-year trends in SAT, SLP, and wind stress based on ensemble members with large positive and negative equatorial wind stress trends (Fig. 4) reveal large differences in the warming trend over regions where SATs are strongly correlated with equatorial winds (Fig. 1). Relative cooling over the central and eastern tropical Pacific and adjacent areas in South America, east Asia, Australia, the western Indian Ocean along with relative warming in the Northwest and the Southwest Pacific are displayed in those ensemble members in which the Pacific trade winds are intensified (Fig. 4b, e, h). The opposite occurs when the Pacific trade winds are reduced (Fig. 4c, f, i).

For each ensemble set, the range in the GMT trend is comparable with the ensemble-mean trend (Supplementary Table 3), again indicating the importance of internal variability on these timescales (Fig. 4a, d, g). As with the historical analysis, there are statistically significant positive correlations between the zonal wind stress trends and GMT trends across ensemble members (Figs. 2, 4) with correlations of 0.57, 0.66, and 0.67 in the CESM-Hind-Proj, the KCM-ICs and Mk3L-ICs, respectively (Fig. 4a, d, g). However, the regressions are different from model to model (Fig. 4a, d, g), implying that the SST response to the tropical winds (i.e., the strength of the wind-SST feedback) is very different across the three models. Given the large relative uncertainty in the GMT 20-year trend in each ensemble (Supplementary Table 3; Supplementary Fig. 6), there is inherently low predictability in the magnitude of the GMT increases on this timescale, unless the Pacific wind trends (and associated IPO phase) can themselves be predicted. In general, the rate of global warming in Mk3L is relatively low compared with that simulated in KCM and CESM1-CAM5 (Fig. 4a, d, g). It was previously shown that Mk3L has a lower transient climate response compared with other climate models, which is mainly owing to parametrization of key radiative feedback processes such as water vapor, lapse rate, snow, and ocean heat uptake in the model^38,45^.

### Incorporating oceanic initial conditions into decadal predictions

Recent work using initialized simulations in a single model^25^ suggests that the state of upper-ocean heat content can provide some predictability around phase transitions of the IPO. Here, we extend this work to examine the potential to improve the predictability of 20-year trends in two distinct model ensembles, given knowledge of the initial phase of the IPO. In particular, we examine climate trends from perturbed atmosphere ensembles starting from a positive and negative IPO state in the KCM and Mk3L models (termed KCM-IPO-Warm, KCM-IPO-Cold, and Mk3L-IPO-Warm, Mk3L-IPO-Cold, respectively).

**Fig. 5:**
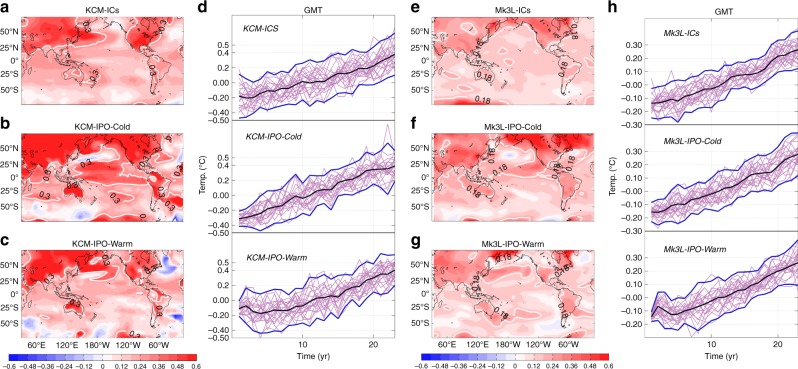
Surface warming in randomly chosen and fixed oceanic initial conditions experiments. Ensemble mean of 20-year trend in SAT (**a**–**c**, **e**–**g**) and time series of the GMT (**d**, **h**) obtained from the KCM-ICs (**a**, **d**), the KCM-IPO-Cold (**b**, **d**), the KCM-IPO-Warm (**c**, **d**), the Mk3L-ICs (**e**, **h**), the Mk3L-IPO-Cold (**f**, **h**) and the Mk3L-IPO-Warm (**g**, **h**) ensemble of global warming simulations. The SAT trends are computed the first 20-year after initialization. **d**, **h** Thin (thick black) lines indicate the ensemble mean and thick blue lines denote ± 2 × standard deviation. The globally averaged SAT time series are relative to its average over the first 20-yr. Please note that **a**–**c** and **e**–**g** have identical color-scale. Contours denote 0.3 and 0.18 °C/decade in **a**–**c** and **e**–**g**, respectively

The connection between the Pacific trade winds and regional changes in temperature and circulation and with global surface warming in these IPO experiments is consistent with previous ensembles used in this study (Fig. 4; Supplementary Fig. 7, Supplementary Fig. 8). Time series shown in Fig. 5 are the GMT in each ensemble member. In each experiment, the GMT is computed relative to its average over the first 20-years. For the first few years, the ensemble spread in GMT in the simulations initiated during the peak of either a positive or negative IPO phase is smaller than that obtained from an ensemble initiated from random ocean states (i.e., KCM-ICs and Mk3L-ICs; Fig. 5d, h). On longer timescales, the ensemble spreads in the annually averaged GMT become very similar across the three ensembles for each model. The rapid increase in the spread of GMT is a result of various factors limiting the climate forecast skill in multi-year to decadal timescales, such as stochastic forcing and strongly nonlinear climate dynamics^46,47^. The rate of global warming, indicated by the ensemble mean, differs across these ensembles (Fig. 5d, h). Namely, when the projections start from the negative phase of the IPO (i.e., KCM-IPO-Cold, Mk3L-IPO-Cold; Fig. 5d, h) the ensemble-mean warming is faster over the first decade than in the randomly initialized ensemble, for both models. The opposite is true for the ensembles started from the positive IPO state (Fig. 5d, h; Supplementary Fig. 9). Indeed, the decadal trend (10-year) in the annual mean GMT in each ensemble derived from the forecast data show significant differences across the experiments. The trend in the ensemble mean of KCM-ICs, KCM-IPO-Cold, and KCM-IPO-Warm is 0.21 ± 0.17, 0.31 ± 0.14, and 0.03 ± 0.14 °C/decade, respectively. The corresponding trends in Mk3L-ICs, Mk3L-IPO-Cold, and Mk3L-IPO-Warm are 0.12 ± 0.08, 0.15 ± 0.04, and 0.08 ± 0.09 °C/decade, respectively (Supplementary Fig. 9). In both models, there is a significant difference (95% confidence interval) in the GMT decadal trends in the ensemble set initiated from the positive IPO and that initiated from the negative IPO. Computing a similar trend as above but starting from the second year of simulation in each ensemble mean (Supplementary Fig. 9) leads to similar conclusion: KCM, Mk3L-IPO-warm experiments display reduced global warming compared with the KCM, Mk3L-IPO-Cold experiments. Thus, despite the presence of the rapidly varying atmospheric forcing and other predictability barriers over the tropical Pacific^46,47^, GMT decadal prediction can be significantly improved by implementing the past climate trajectory in the model. This essentially demonstrates that knowledge of the oceanic initial conditions has the potential to improve the accuracy of near-term GMT projections over a decadal timescale.

On a 20-year timescale, the difference in the rate of global warming across model ensembles seems to be less consistent (Supplementary Fig. 9). For example, in the KCM ensembles, 20-year global warming in the ensemble set initialized from the positive IPO (KCM-IPO-Warm) is approximately the same as that for the randomly chosen ICs ensemble (KCM-ICs; ~0.43 °C) although faster warming is indeed simulated in the simulation initiated from the negative IPO (0.67 °C; Supplementary Fig. 9). In contrast, Mk3L ensembles show almost the same distribution of warming over the first 20-years (Supplementary Fig. 9) regardless of initial conditions. Thus, the state of the IPO affects predictions of GMT on a 10-year timescale, but shows weaker importance at longer timescales as greenhouse effect dominates.

Why the IPO state could significantly affect GMT prediction might come down to the fact that the IPO is a quasi-steady process (i.e., stationary time series) with tendency to revert toward its climatological mean over a ~20-year timescale. As such, the subsequent 20-year trend after any maximum IPO phase is very likely to be negative. This basically leads to a tendency for decadal cooling over the tropical Pacific and a decline in GMT following the maximum positive IPO phase. The opposite is true after minimum IPO phases. This notion is supported by composite analysis based on IPO extremes ( ±1 SD) in the control runs (Supplementary Fig. 10). In general, 20-year trends in GMT after maximum IPO cold phases tend to be large and positive, while these trends tend to be large and negative after IPO warm phases (Supplementary Fig. 10, b, e, h). Indeed, the spatial structures of the SAT trend obtained from the composite analysis are very similar to the IPO patterns (Supplementary Fig. 10,c,f,i), further supporting this mechanism for drivers of decadal variability in GMT.

How the state of the IPO could lead to such decadal trends of GMT is not fully understood. As shown in previous studies^1,4,6^, IPO cycles have a critical role in redistribution of heat storage through changes in the equatorial Ekman divergence over the equatorial Pacific, which is accompanied by anomalous poleward surface heat transport to the mid and high latitudes. Another potential mechanism may be through altered incoming shortwave radiation, particularly since the GMT and globally averaged net shortwave radiation are strongly correlated^48^. Specifically it could be that incoming solar radiation is reduced following the IPO-Warm owing to increasing cloud cover, thus leading to decreased GMT. Preliminary analysis with the KCM control run shows that such tendency exists but somewhat weak (not shown). Further study is needed to clarify the pertinent mechanisms, which are beyond the scope of this paper.

The spatial structures of ensemble-mean 20-year SAT trends are noticeably different across the different cases of initial conditions for each of the models (Fig. 5a–c, e–g). Compared with the ICs trends in both models IPO-Cold trends display larger warming over the central tropical and subtropical Pacific and adjacent continents, North America, Asia and relative cooling over the Northwestern Pacific (Fig. 5). The opposite behavior is obtained in the IPO-Warm trends in both models (Fig. 5). Over large parts of the basin and adjacent continent, the difference between the KCM-IPO-Cold and the KCM-IPO-Warm is statistically significant (at 95% confidence level), which is similar to results obtained from the composite analysis based on IPO maxima and IPO minima in the control runs (Supplementary Fig. 10). These spatial structures are reminiscent of the positive phase of the IPO (Supplementary Fig. 11a). A similar structure, but smaller magnitude, is obtained from the difference between the Mk3L-IPO-Cold and the Mk3L-IPO-Warm ensemble sets (Supplementary Fig. 11b). In addition, most members, ~17 (14) out of 22, in the KCM-IPO-Cold (the Mk3L-IPO-Cold) ensemble set display an IPO-positive-like pattern in the first two decades of integration, whereas the IPO-positive-like and the IPO-negative-like patterns are rather equally likely in the KCM-ICs (the Mk3L-ICs; see Supplementary Fig. 12-S18). Roughly opposite patterns were simulated when the simulations started from the positive phase of the IPO (see Supplementary Fig. 14, S17, S18). Thus, on regional scales, the state of the IPO appears to have significant influences on the magnitude of 20-year SAT trends over large parts of the Pacific and adjacent continents.

Thus, our results suggest that taking into account the state of oceanic conditions associated with the IPO could hold the potential to narrow down uncertainty in multi-decadal regional SAT projections. However, owing to the nonlinear nature of the climate system and large exchanges of heat and momentum between the ocean and atmosphere, combined with the stochastic nature of the atmosphere, this uncertainty reduction might be strongly state-dependent. Future studies aiming to provide more precise estimates of the associated uncertainties would be beneficial to the climate prediction community. This will likely involve even larger ensembles than considered here, across a diverse but coordinated set of model experiments.

**Fig. 6:**
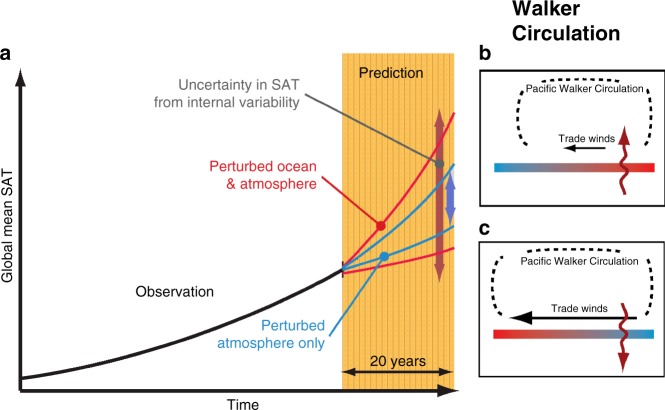
Schematic of the range in simulated global mean temperature trajectories due to tropical Pacific climate variability. **a** The black curve refers to the observed GMT warming in response to increased radiative forcing. Blue (red) curves in **a** denote the projected GMT when the observed oceanic state is used (not-used) to initialize the subsequent climate projections. Corresponding red and blue arrows in **a** indicate the level of uncertainty for each case. Shown in **b**–**c** are the scenarios wherein accelerated (**b**) and reduced (**c**) GMT warming is often associated with anomalous Pacific trade winds (and thus Walker Circulation strength) and corresponding anomalous heat fluxes between the ocean and atmosphere

To summarize, we have analyzed several ensembles of climate change simulations across three fully coupled climate models, subject to different external forcing and initial states. Our goal was to examine and quantify the uncertainty in estimates of forced globally averaged SAT trends in hindcast and projection simulations associated with internal variability and the associated regional signatures. In the presence of identical external forcing, a large spread in 20-year tropical and subtropical Pacific trends was found, in each model ensemble, arising from internal climate variability. We showed that in each ensemble set, the rate of global warming over a 20-year timescale is closely linked to tropical Pacific internal climate variability, whereby intensified trade winds, as a good representative of tropical Pacific climate, are associated with reduced globally averaged warming, and weaker trade winds with accelerated global warming (Fig. 6). The range of GMT 20-year trends sometimes exceeds the ensemble-mean warming trend in each ensemble set. We further showed that, on decadal or even multi-decadal timescales, there is the potential to narrow this range if the observed ocean state is known and used to initialize the model forecasts (Fig. 6). Furthermore, our findings indicate that when the model integration starts from a negative (positive) phase of the IPO, the subsequent decade is more likely to exhibit accelerated (reduced) global warming. This suggests that there is an increased likelihood of accelerated global warming in the coming decade since we have, in recent years, been in a strongly negative IPO state^4^. Our idealized experiments support the use of initialized climate model projections for making decadal predictions. Our findings nonetheless suggest that internal variability can potentially obscure the forced signal in the tropical Pacific sector and in the GMT on decadal timescales. However, the relative importance of this will decline as atmospheric GHG concentrations continue to increase into the future.

## Methods

### Model experiments

To quantify the uncertainty in tropical Pacific climate projections owing to internal climate variability, we examine multiple realizations (ensemble members) from a number of large ensemble experiments from different climate models. Ensemble members from each experiment are subject to identical external forcing but are started from different initial conditions. Our analysis is based on three different fully coupled climate models (Supplementary Table 1): namely the Kiel Climate Model^49^ (KCM), CSIRO-Mk3L^45,50^, and CESM1-CAM5^51^. These models employ different physical parameterizations and numerical schemes and have different ocean and atmosphere resolutions, which allow us to assess the robustness of the results to structurally different models.

We perform a millennial timescale control run with the KCM at approximately present-day CO_2_ levels (348 ppm). Branching off from this control simulation we undertake three 22-member ensemble 100-year global warming experiments based on idealized transient radiative forcing according to a 1% per year (p.a.) CO_2_ warming scenario^44,52^. In the first ensemble set, integrations start from different oceanic and atmospheric initial conditions (ICs) at different times of the control run. These initial conditions cover a broad range of climate regimes (Fig. 1a). This ensemble is termed KCM-ICs. The ICs indicates the method of the ensemble generation. In the second and the third ensemble set, the ensemble spread is produced by only perturbing the atmospheric initial conditions while the oceanic initial conditions remain fixed (i.e., from a single point in the control simulation; Fig. 1a). In this case, any spread is owing to atmospheric variability and nonlinear climate dynamics. In the second ensemble, termed as KCM-IPO-Cold, the oceanic initial state corresponds to negative phase of the IPO, whereas it matches the positive phase in the third ensemble, termed as KCM-IPO-Warm (Fig. 1a). Comparison between the ICs and the latter ensemble sets allows us to estimate the contribution of the IPO initial state to the GMT projections and to isolate the uncertainty related to chaotic atmospheric fluctuations from those owing to the oceanic initial state^44,53^.

For the CSIRO-Mk3L, we undertake a 1100-year control run with constant CO_2_ at preindustrial levels (280 ppm). Again, a 22-member ensemble with 1% p.a. increasing CO_2_ is performed with a similar configuration to the KCM ensemble sets; i.e., with different oceanic and atmospheric initial conditions (termed Mk3L-ICs; Supplementary Table 1) and fixed oceanic initial conditions in negative (termed Mk3L-IPO-Cold) and positive (termed Mk3L-IPO-Warm) phases of the IPO (Fig. 1c; Supplementary Table 1).

We also analyze output from CESM1-CAM5 experiments. For this model a 1800-year control run at preindustrial CO_2_ concentration (280 ppm) was integrated. A single realization was integrated for 71 years with the external forcing according to that observed from 1850 to 1920. Thirty-five global warming experiments are undertaken by perturbing atmospheric initial conditions with the same oceanic state at 1920. They are then subject to historical radiative forcing from 1920 to 2005 and are extended according to the Representative Concentration Pathway 8.5^52^ scenario until 2100. This ensemble set is termed CESM1-Hind-Proj. Hind and Proj here refer to hincast and projection, respectively.

The KCM and Mk3L global warming simulations (with 1% CO_2_ warming scenario) were performed as part of this project. The long control simulations predated this project (which is the reason for them being subject to different greenhouse levels representative of present day and preindustrial conditions). The CESM runs are pre-existing simulations that have been made available to the community^51^; however, only the Representative Concentration Pathway 8.5 projections are provided.

### Observations

The model results are compared to observational reanalysis products. For SAT, we use the gridded reanalysis from the Goddard Institute for Space Studies (GISS)^54^, and for wind stress and SLP we utilize the interim European Centre for medium-Range Weather Forecast (ECMWF) Re-Analysis (ERA-Interim)^55^. We also use SST observations from HadISST^56^ to evaluate the areal averaged of SST over Niño3.4 region (5°N–5°S, 170°W–120°W).

### Metrics

The strength of the Pacific trade winds is estimated by taking the area average of zonal wind stress over the western and central tropical Pacific (160°E–150°W, 5°S–5°N) where the trade wind anomaly pattern shows the most pronounced interannual to multi-decadal variability^57^. To examine the link between the IPO and the trade winds, we use the TPI as a proxy for the IPO^35^. This index is defined as the difference between the SST anomaly over the central tropical Pacific (10°S–10°N, 170°E–90°W) and the average of the SST anomalies over the Northwest (25°N–45°N, 140°E–145°W) and Southwest Pacific (50°S–15°S, 150°E–160°W). These regions are shown with rectangular boxes in Fig. 1d.

To reduce the impacts of the long-term spurious climate drift on our results, linear trend simulations was subtracted from control runs^58,59^. To estimate the range of uncertainty in the GMT projections on different timescales, which result from internal variability alone, we utilized moving trends (with a window size ranging from 5 to 35 years) on annually averaged GMT control run time series. For each window size, the 1 and 99 percentile range in ensemble trends is used to quantify the uncertainty owing to internal variability.

Historical uncertainty in the 20-year climate simulations is estimated by analyzing the periods corresponding to 1992–2011 in the CESM-Hind-Proj ensemble set. The uncertainty is defined as the spread in the 20-year trends across the model ensemble members. In regard to future climate projections, the uncertainty is estimated from the 20-year period corresponding to 2016–2035 in the CESM-Hind-Proj and the first 20-year period of simulation in the other ensembles.

## Supplementary information

Supplementary Information

## Data Availability

Yearly mean gridded data from the KCM and the Mk3L simulations are available through https://data.geomar.de/thredds/catalog/open_access/bordbar_et_al_2019_nc/catalog.html and citable using the following 10.1038/NCLIMATE2569, 10.1002/2016GL072355, 10.1175/JCLI-D-11-00287.1, 10.1175/JCLI-D-12-00108.1. Simulated data from the CESM1-CAM5 experiments can be downloaded from http://www.cesm.ucar.edu/experiments/cesm1.1/LE/.

## Code availability

Data processing codes are available from the corresponding author upon request.
