# Supplementary material for: Uncertainty in near-term global surface warming linked to tropical Pacific climate variability
Source: Nat Commun. 2019 Apr 30;10:1990. doi: 10.1038/s41467-019-09761-2 (PMC6491465; doi:10.1038/s41467-019-09761-2)
Supplement: Supplementary file 1 — Supplementary Information [file 41467_2019_9761_MOESM1_ESM.pdf]

Supplementary Material for

Uncertainty in near-term global surface warming linked to tropical Pacific climate variability

Mohammad Hadi Bordbar*[1], Matthew H. England[2], Alex Sen Gupta[2], Agus Santoso[2,3], Andréa S. Taschetto[2], Thomas Martin[1], Wonsun Park[1], Mojib Latif[1,4]

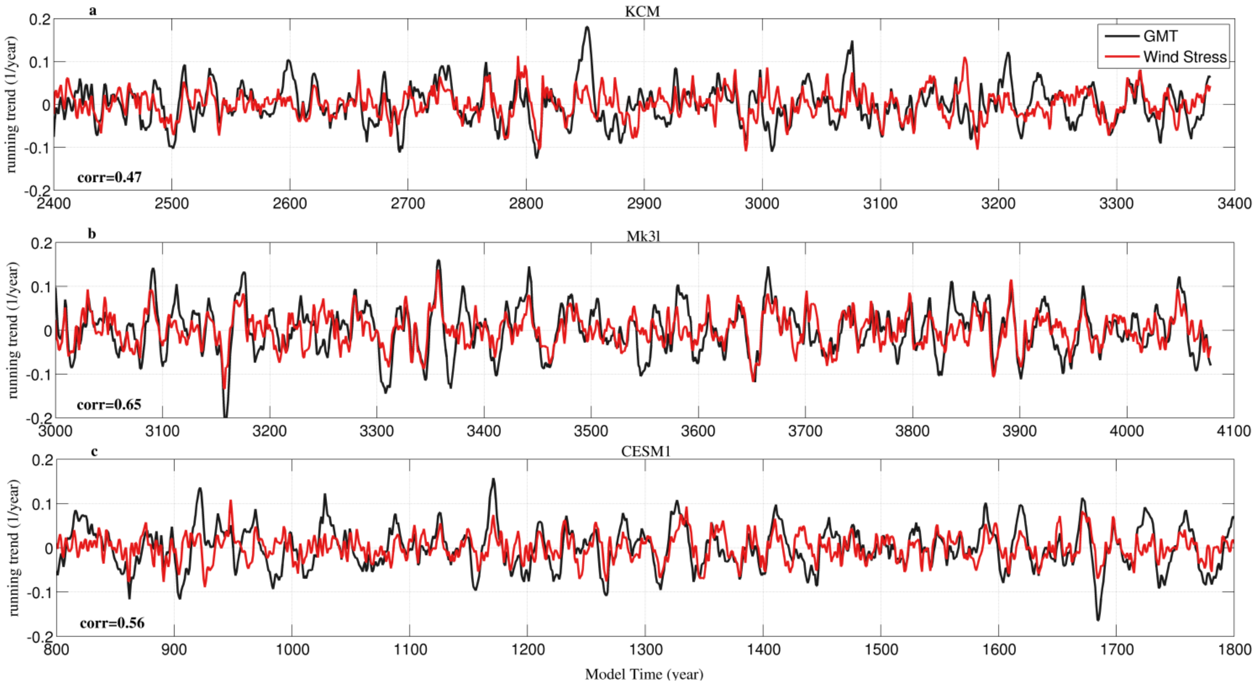

Supplementary Figure 1: Time series of Pacific trade winds and globally averaged surface air temperature. Shown are the time series of 21-year running trend of zonal wind stress over the western equatorial Pacific (red solid lines) and globally averaged SAT (black solid lines) obtained from the KCM (a), the Mk3l (b) and the CESM-CAM5 (c) control runs. Prior to computing the trends, all time series have been normalised. The corresponding correlation is shown in the bottom left corner of each panel.

[1]GEOMAR Helmholtz Centre for Ocean Research Kiel, D-24105 Kiel, Germany [2]Climate Change Research Centre and ARC Centre of Excellence for Climate Extremes, University of New South Wales, New South Wales 2052, Australia [3]Centre for Southern Hemisphere Oceans Research (CSHOR), CSIRO Oceans and Atmosphere, Hobart 7004, Australia [4]Excellence Cluster "The Future Ocean", University of Kiel, D-24098 Kiel, Germany
*e-mail: mbordbar@geomar.de, Ocean.Circulation@gmail.com

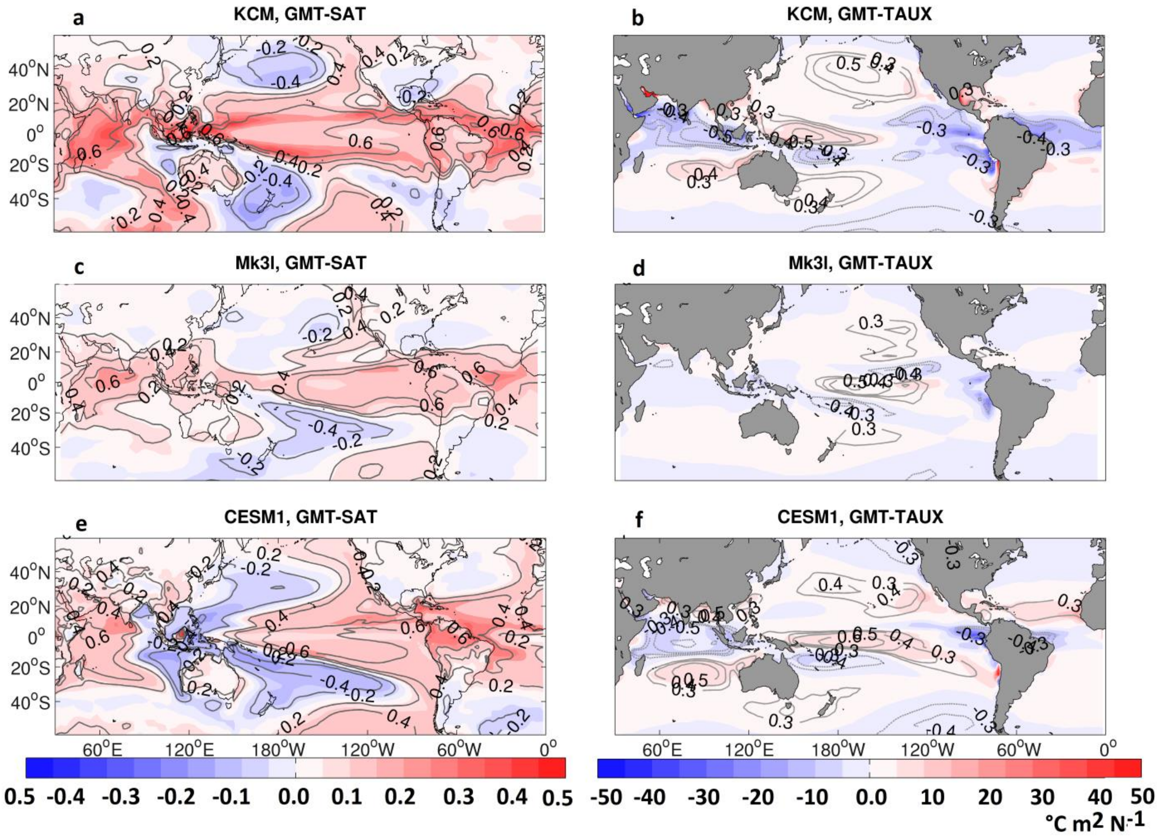

Supplementary Figure 2: Correlation between surface air temperature, zonal wind stress and global mean temperature. Regression coefficients (color shading) between annually averaged GMT and SAT (left panels) and GMT and zonal wind (right panels; °C m^2 N^{-1}) obtained from the KCM, Mk3l and CESM1 control runs. Contours show the corresponding correlation.

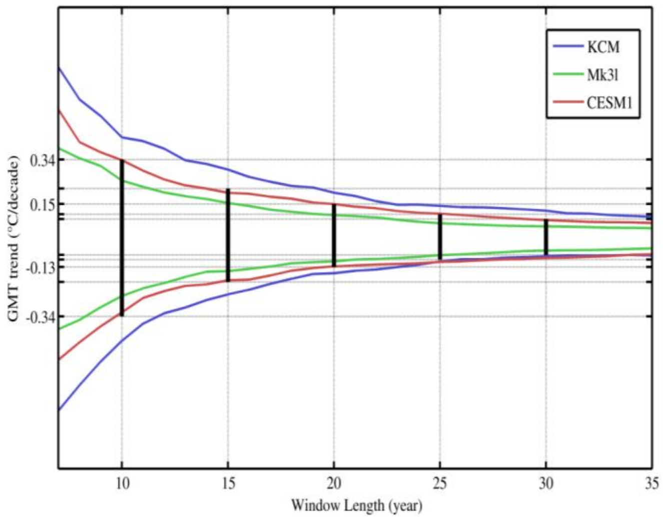

Supplementary Figure 3: Range of linear trends in global mean temperature over different time-scales. Upper and lower limits of linear trends in the GMT in different time scale which are computed from the KCM (blue solid line), the Mk3l (green solid line) and the CESM1 (red solid line) control runs. For each time scale, the running trend is computed over the entire period of the control run and the lower (upper) limit is defined as the 1st (99th) percentile of the running trend. Vertical black lines denote the range of 10, 15, 20, 25, 30-year trends calculated from the difference between mean of the upper and lower limits.

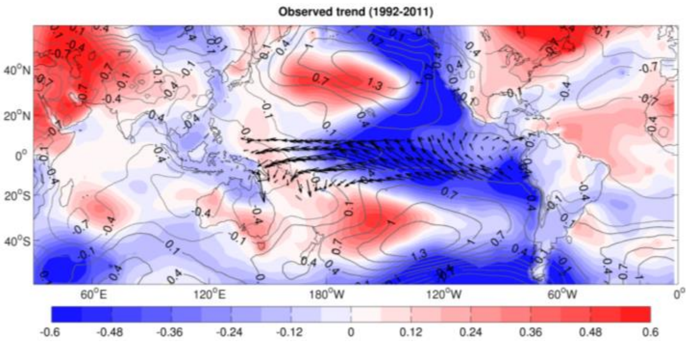

Supplementary Figure 4: Linear trend in observed SLP, SST and wind stress during 1992-2011. Shown are the 20-year trends in the observed SAT (shading,°C/decade), SLP (contours, hPa/decade) and zonal wind stress (vectors, N m^2/decade) during 1992-2011. The globally averaged SAT trend has been subtracted from each grid point. The observed wind stress and SLP are taken from ERA-Interim Reanalysis and SAT is from Goddard Institute for Space Studies (GISS) climatology.

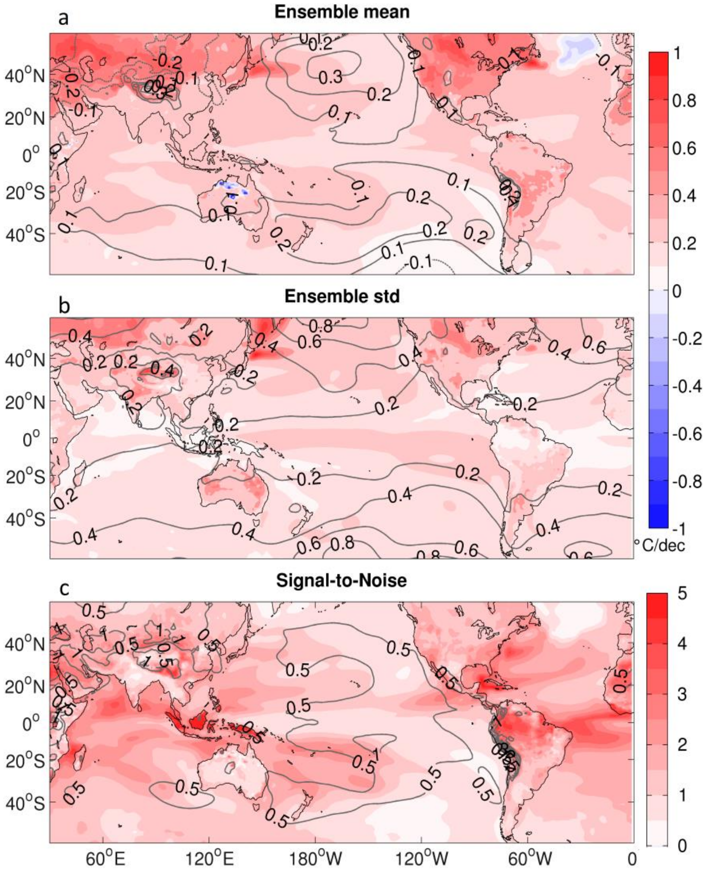

Supplementary Figure 5: Ensemble mean and spread of 20-year trends in SST and SLP. The ensemble mean (a), ensemble spread (b) and signal-to-noise ratio of 20-year trend in SAT (shading; °C/decade) and SLP (contours; hPa/decade) obtained from CESM-Hind-Proj global warming experiments. The trend corresponds to the period of 1992-2011. The linear trend is computed for each ensemble member at each grid point. The ensemble spread is represented by the standard deviation of the trend. The signal-to-noise is the ratio between the magnitude of ensemble mean trend and the ensemble spread. Consistent with previous studies[1,2], this figure indicates that in regional scale periods longer than two decades is required to detect anthropogenic climate change signals from the internal variability.

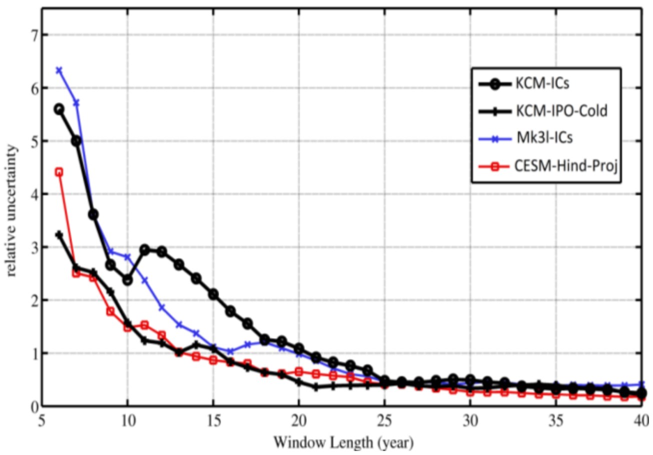

Supplementary Figure 6: Uncertainty in projected global mean temperature over different time-scales. Relative uncertainty in the GMT linear trends of different durations, from the KCM-ICs, KCM-IPO-Cold, Mk3l-ICs and CESM-Hind-Proj ensemble of global warming simulations. In each duration, first the trend in the GMT of each individual member was computed. Relative uncertainty is defined as the ratio between the range and the ensemble mean of trends across all ensemble members. In each realization and each time scale, the initial year of the trend in the KCM-ICs, KCM-IPO-Cold, Mk3l-ICs are the starting year of integration whereas it is the year 2016 in the CESM-Hind-Proj.

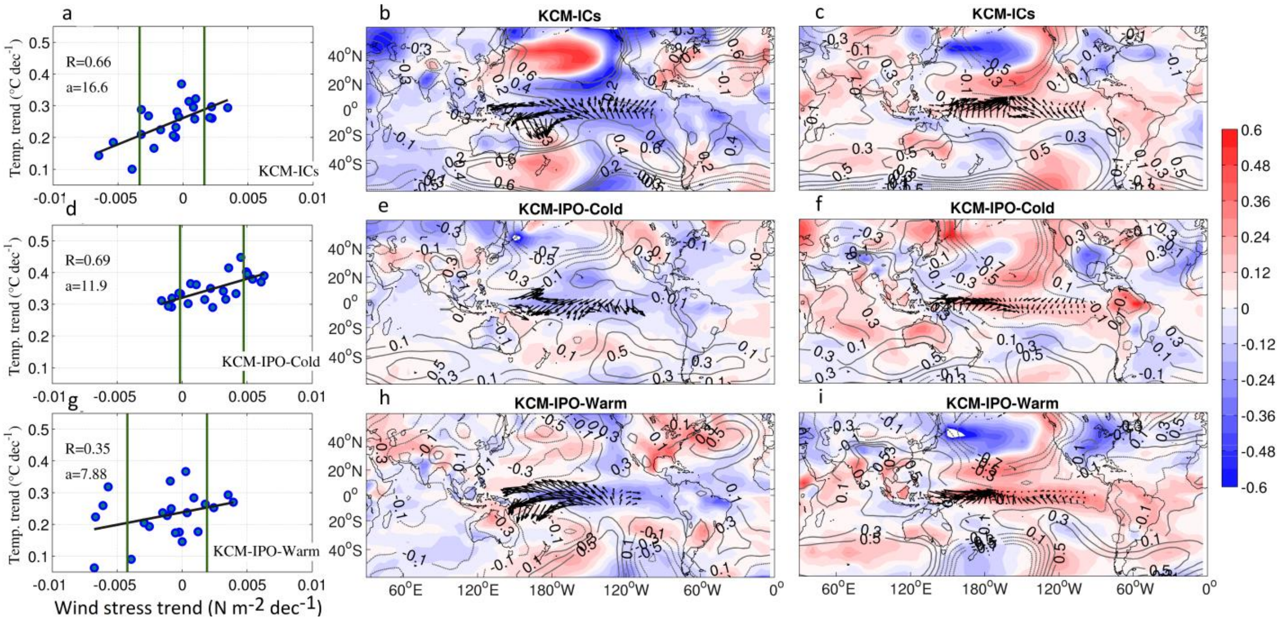

Supplementary figure 7: Composites of 20-year trends in SAT, SLP, and Pacific trade winds in three ensembles of a global warming simulation. Scatter plot of 20-year trends in the zonal wind stress (N m^{-2} dec^{-1}) and globally averaged SAT (°C dec^{-1}) from individual members of KCM-ICs (a), KCM-IPO-Cold (d) and KCM-IPO-Warm (g) ensemble of global warming experiments. The vertical solid lines indicate the mean±Std of the trend in the wind stress and black solid lines denote the regression lines between GMT and wind stress trends. The correlation between the wind stress and GMT trends and the slope of the regression lines are shown with R and a in the left panels. Other panels show composites of 20-year trends in SAT (shading; °C dec^{-1}), SLP (contours; hPa dec^{-1}) and wind stress (arrows; Nm^{-2} dec^{-1}) based on ensemble members with wind stress trends less than (b,e,h) or exceeding than (c,f,i) 1Std from the KCM-ICs (b,c), the KCM-IPO-Cold (e,f) and the KCM-IPO-Warm (h,i) ensemble of global warming simulations. All trends are related to the first 20-year of the realizations. The corresponding ensemble mean SAT trend is subtracted from each grid point in the middle and right panels.

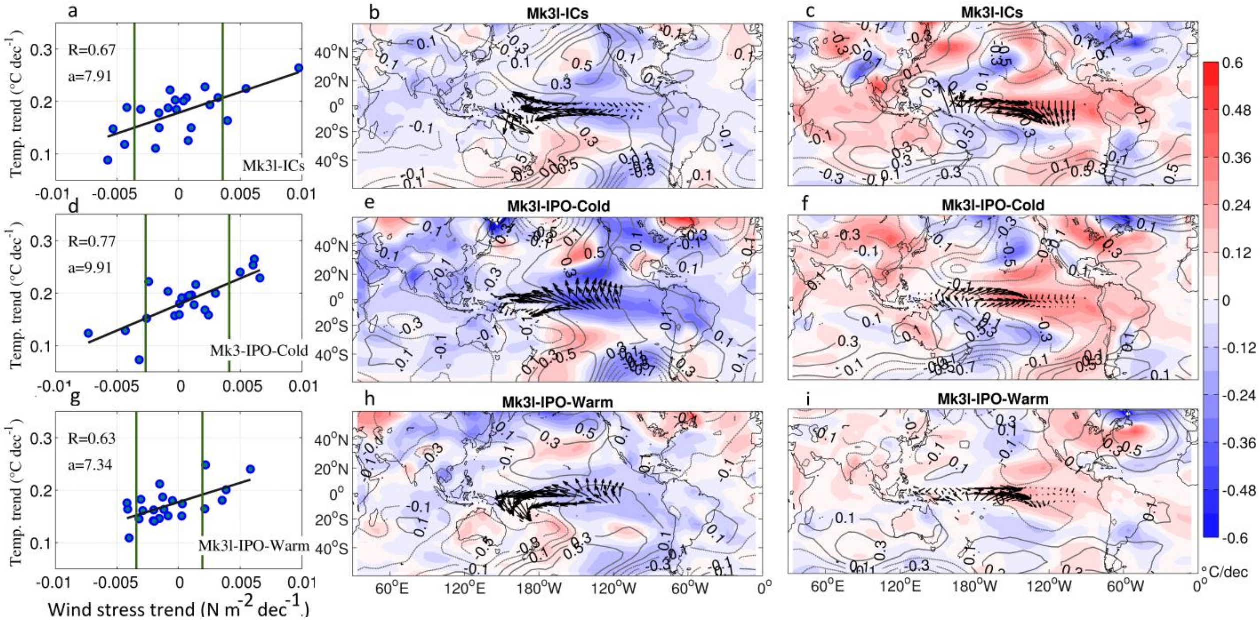

Supplementary figure 8: Composites of 20-year trends in SAT, SLP, and Pacific trade winds in three ensembles of a global warming simulation. Scatter plot of 20-year trends in the zonal wind stress (N m^{-2} dec^{-1}) and globally averaged SAT (°C dec^{-1}) from individual members of Mk3l-ICs (a), Mk3l-IPO-Cold (d) and Mk3l-IPO-Warm (g) ensemble of global warming experiments. The vertical solid lines indicate the mean±Std of the trend in the wind stress and black solid lines denote the regression lines between GMT and wind stress trends. The correlation between the wind stress and GMT trends and the slope of the regression lines are shown with R and a in the left panels. Other panels show composites of 20-year trends in SAT (shading; °C dec^{-1}), SLP (contours; hPa dec^{-1}) and wind stress (arrows; Nm^{-2} dec^{-1}) based on ensemble members with wind stress trends less than (b,e,h) or exceeding than (c,f,i) 1Std from the Mk3l-ICs (b,c), the Mk3l-IPO-Cold (e,f) and the Mk3l-IPO-Warm (h,i) ensemble of global warming simulations. All trends are related to the first 20-year of the realizations. The corresponding ensemble mean SAT trend is subtracted from each grid point in the middle and right panels.

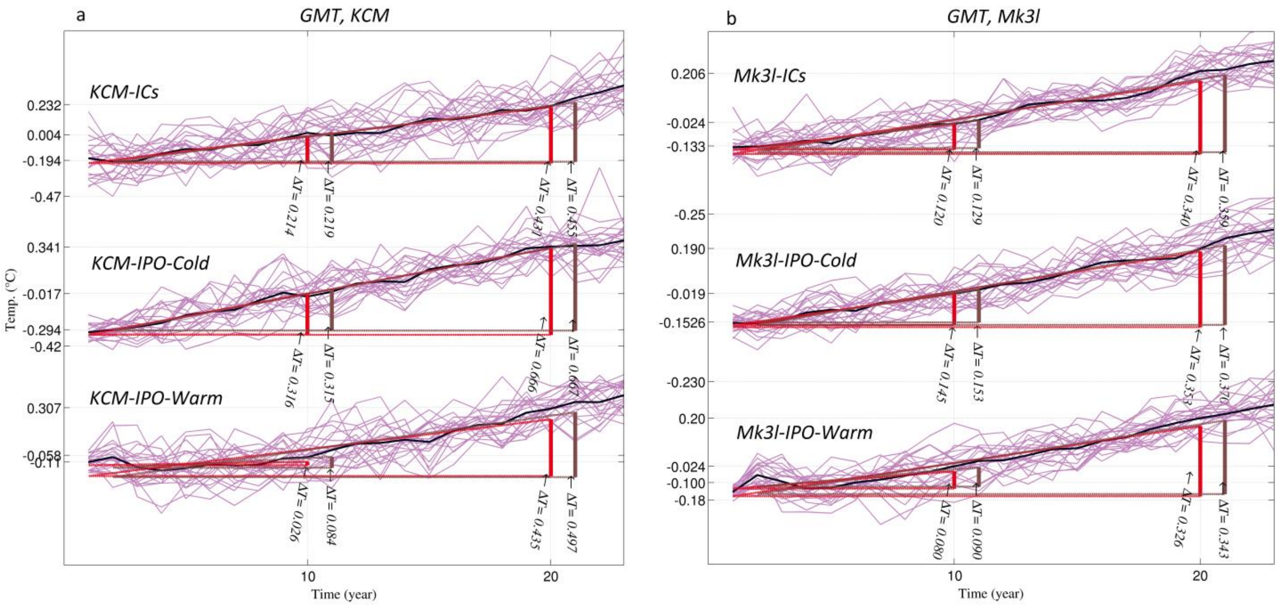

Supplementary Figure 9: Time series of surface warming in randomly-chosen and fixed oceanic initial conditions experiments. Time series of the GMT obtained from the KCM and (a; KCM-ICs, KCM-IPO-Cold, KCM-IPO-Warm) the Mk3l (b; Mk3l-ICs, KCM-IPO-Cold, KCM-IPO-Warm) ensemble of global warming simulations. Thin (thick black) lines indicate individual members (ensemble mean). The globally averaged SAT time series are relative to its average over the first 20-yr. In each ensemble, the trend lines in red (dark green) indicate the trend starting from the first (second) year fitted to the ensemble mean. "ΔTs" indicate the global warming in 10-and 20-year durations which are obtained from the linear trends. Please note that the trends are computed from forecast data only.

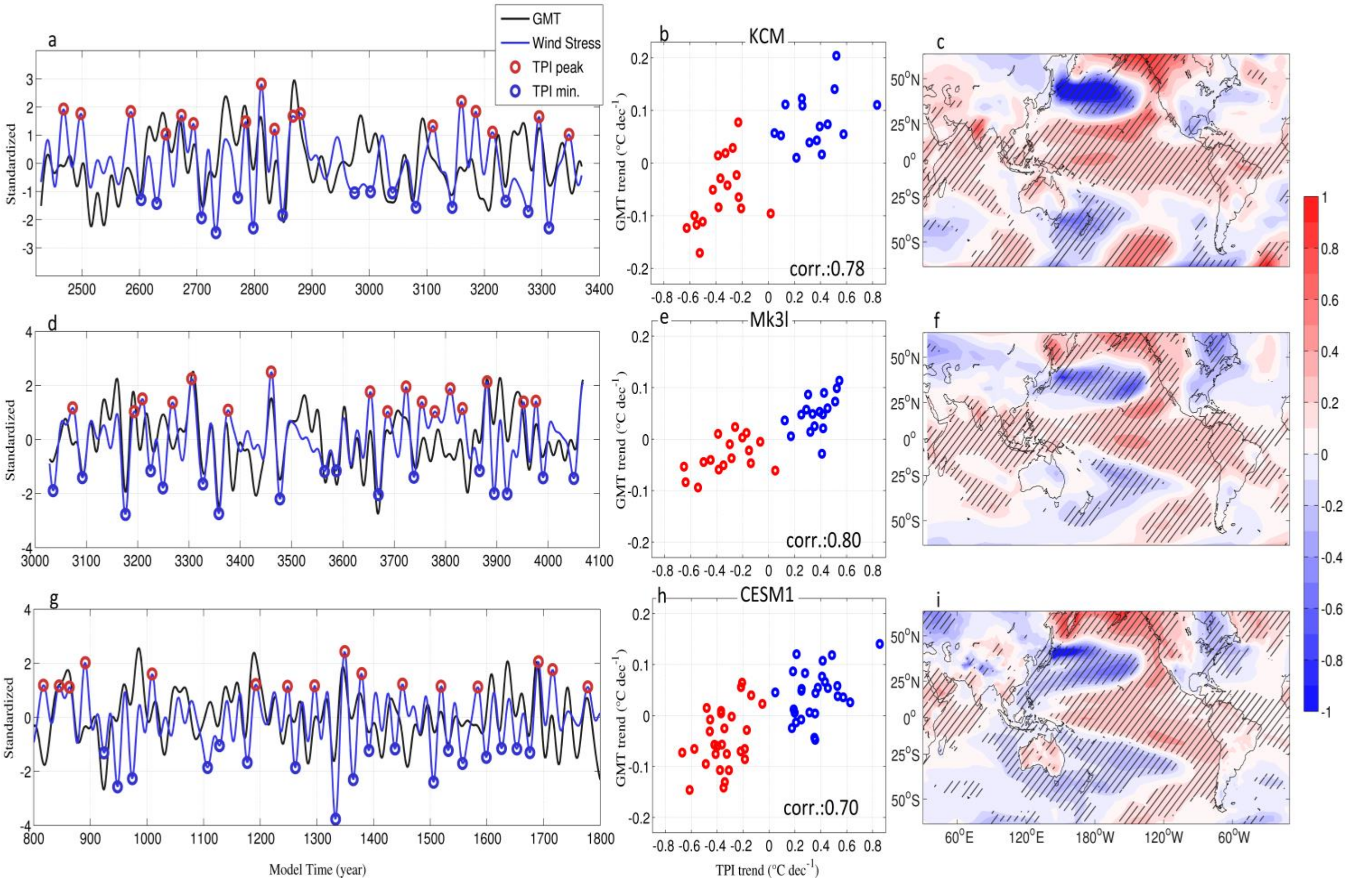

Supplementary Figure 10: Composites of 20-year trends in global mean and local surface air temperature. Time series of low-frequency fluctuations (11-yr low passed filter) in (a,d,g) the GMT (black solid lines) and the Tripole Index (TPI; blue solid lines) from the KCM (a), the Mk3l (d) and the CESM-CAM5 (g) control runs (all time series have been standardised). Red and blue circles in (a,d,g) represent anomalous TPI maxima and TPI minima (± 1 standard deviation), respectively. The scatterplots in (b,e,h) display the trend in the TPI (°C/decade) versus the GMT (°C/decade) over 20-year periods following the TPI maxima (red) and the TPI minima (blue). Corresponding composite spatial pattern of SAT trends obtained from the 20-year periods following the TPI minima minus those following the TPI maxima are shown in (c,f,i). The hatching in (c,f,i) indicates the statistically significant differences between means at the 95% confidence level (according to the student's t test).

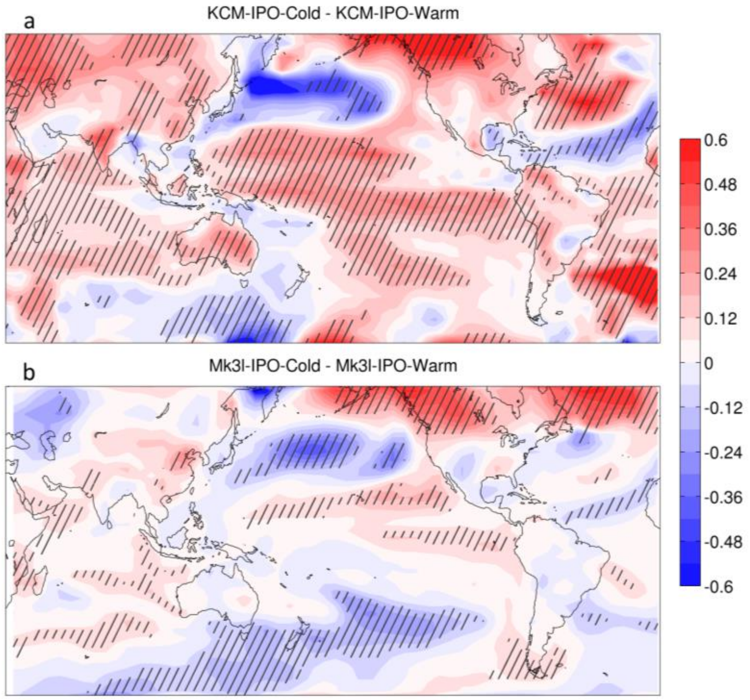

Supplementary Figure 11: Difference between simulated 20-year trends in surface air temperature in IPO cold and IPO warm phases. Top panel (a) show the difference between the ensemble-mean 20-year SAT trend in the KCM-IPO-Cold (Fig. 5b) and that for the KCM-IPO-Warm (Fig. 5c). The bottom panel (b) is as top panel (a) but for the Mk3l-IPO-Cold (Fig. 5h) and Mk3l-IPO-Warm (Fig. 5i). The shown spatial patterns are reminiscent of the positive phase of the IPO. The hatching indicates the statistically significant differences between means at the 95% confidence level (according to the student's [illegible]

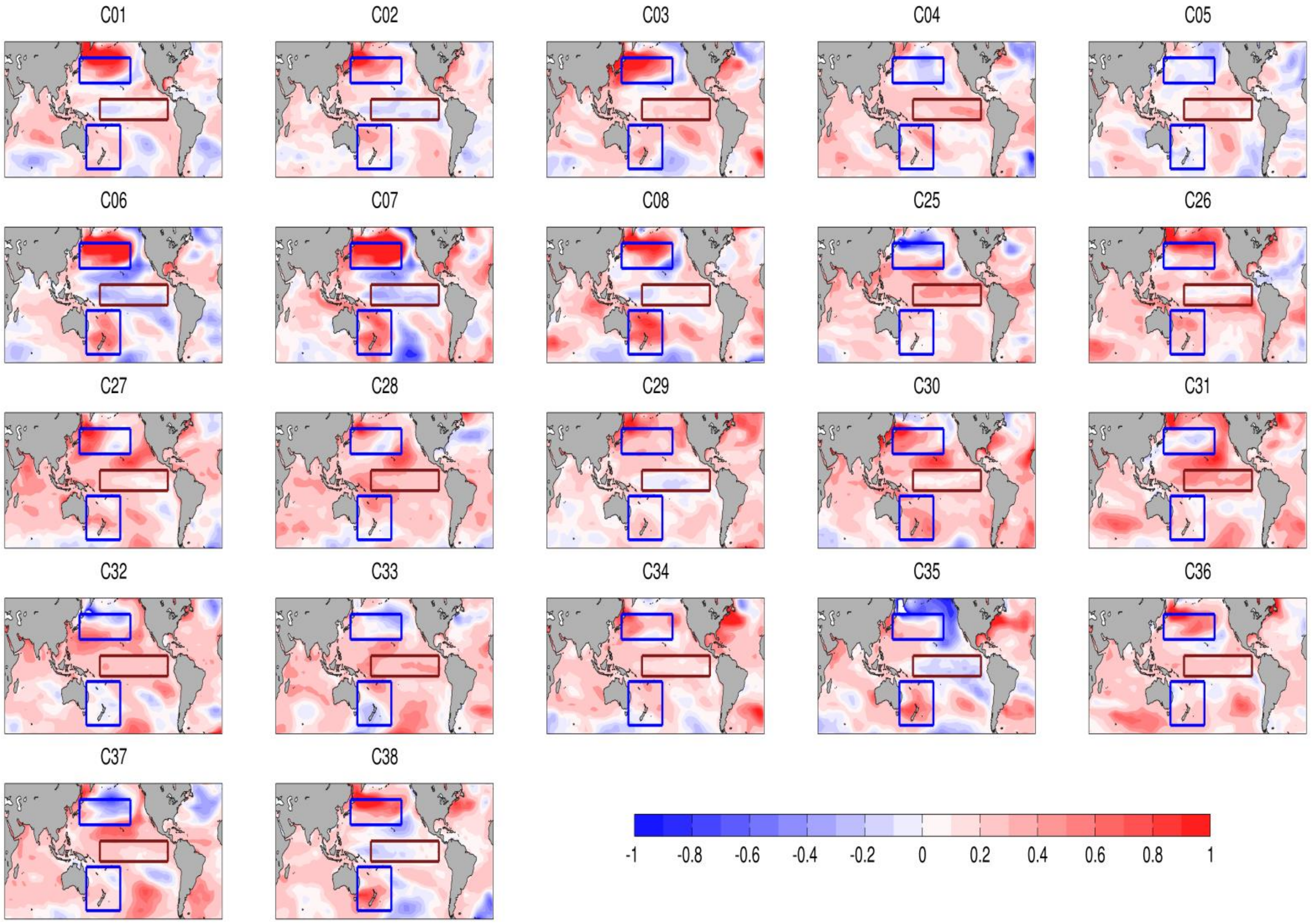

Supplementary Figure 12: Distribution of 20-year trends in surface air temperature in individual KCM ensemble members. 20-yr trend in the SAT (°C dec^{-1}) obtained from individual members of KCM-ICs global warming simulations. In each panel the title shows the name of individual members. Trends are computed from the first 20 year of each simulation. In each panel, rectangular boxes indicate locations used for the calculation of the TPI (Method).

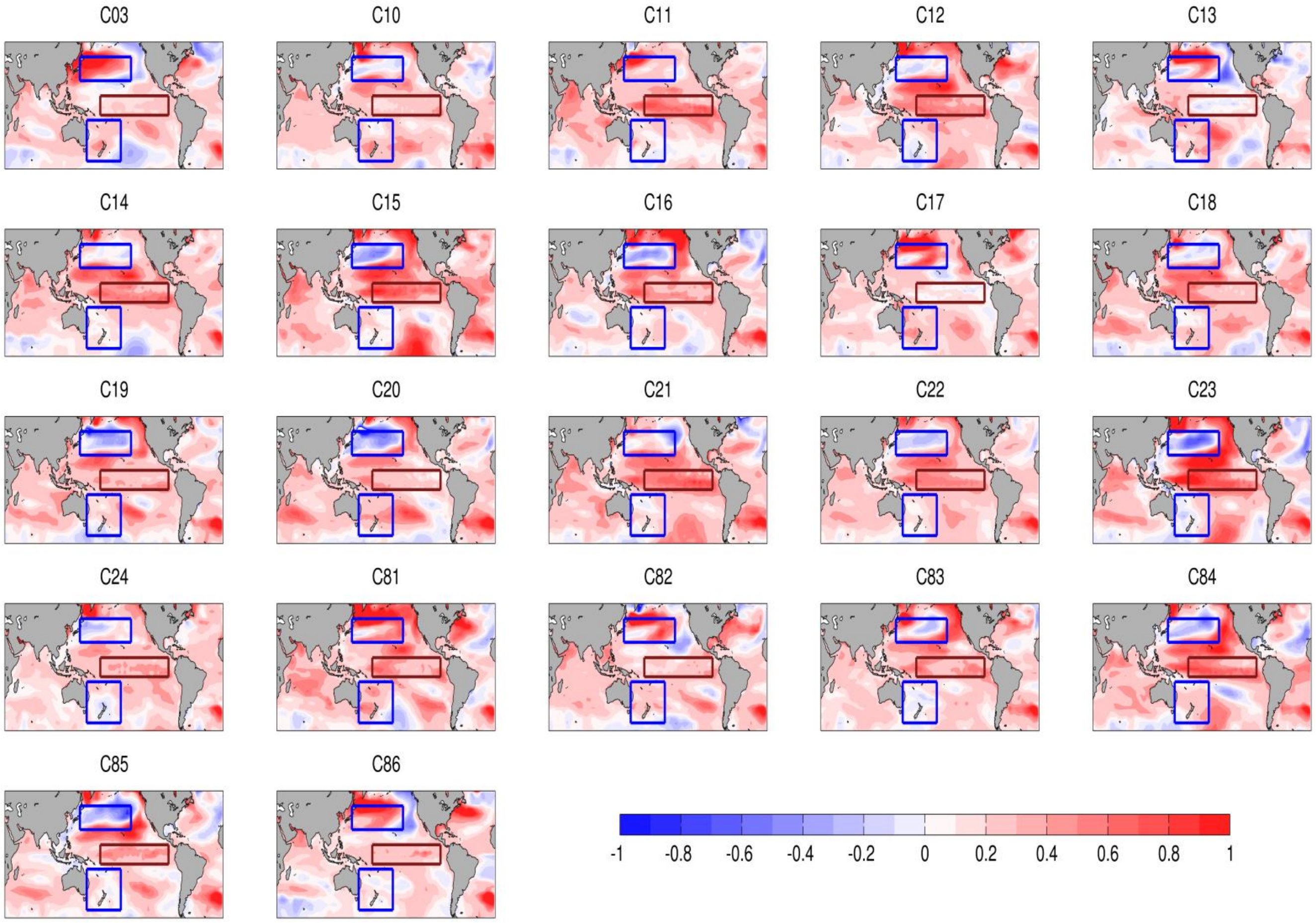

Supplementary Figure 13: 20-year trend in surface air temperature. As in Fig. S13, but for the KCM-IPO-Cold.

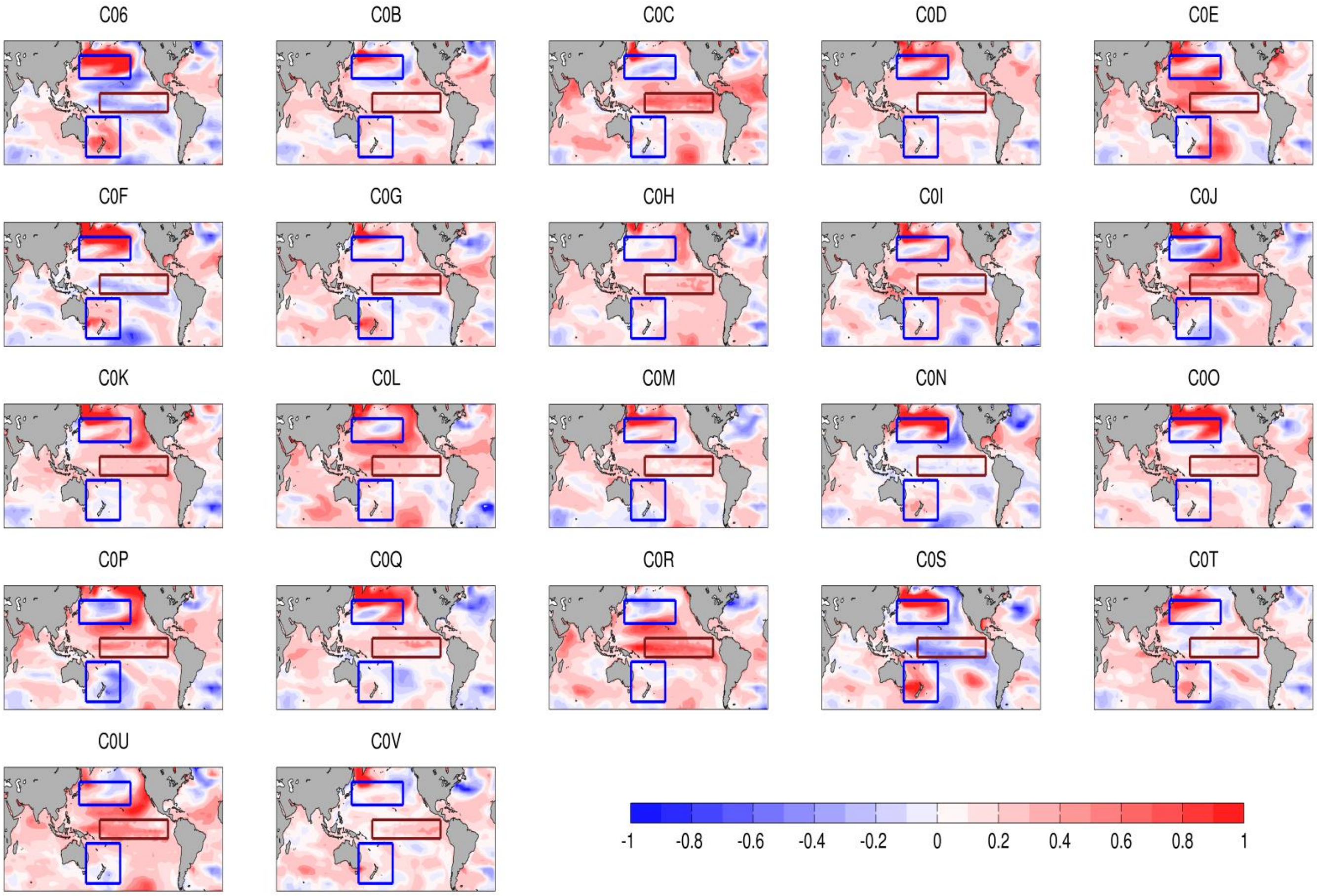

Supplementary Figure 14: 20-year trend in surface air temperature. As in Fig. S13, but for the KCM-IPO-Warm.

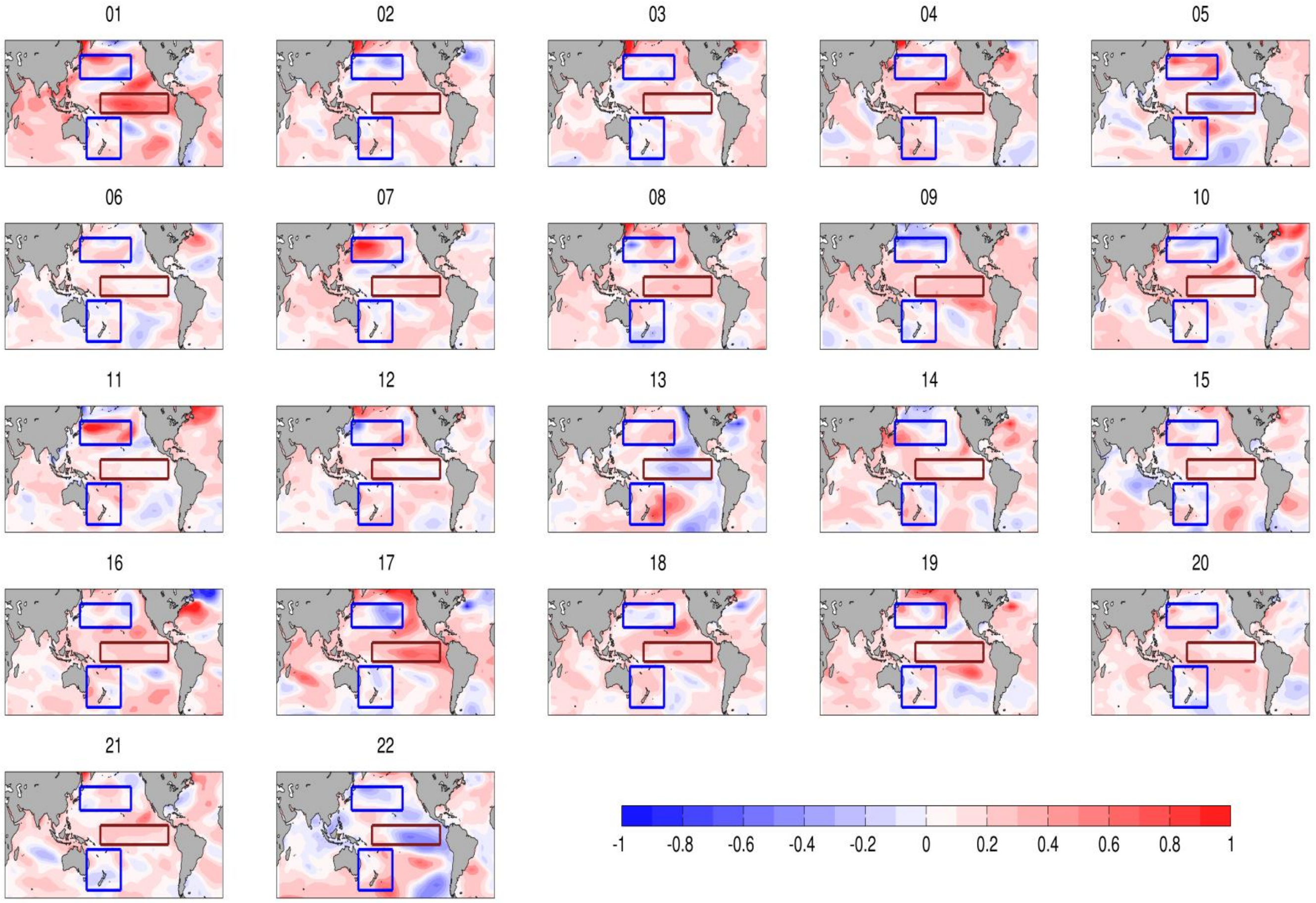

Supplementary Figure 15: 20-year trend in surface air temperature. As in Fig. S13, but for the Mk3l-ICs.

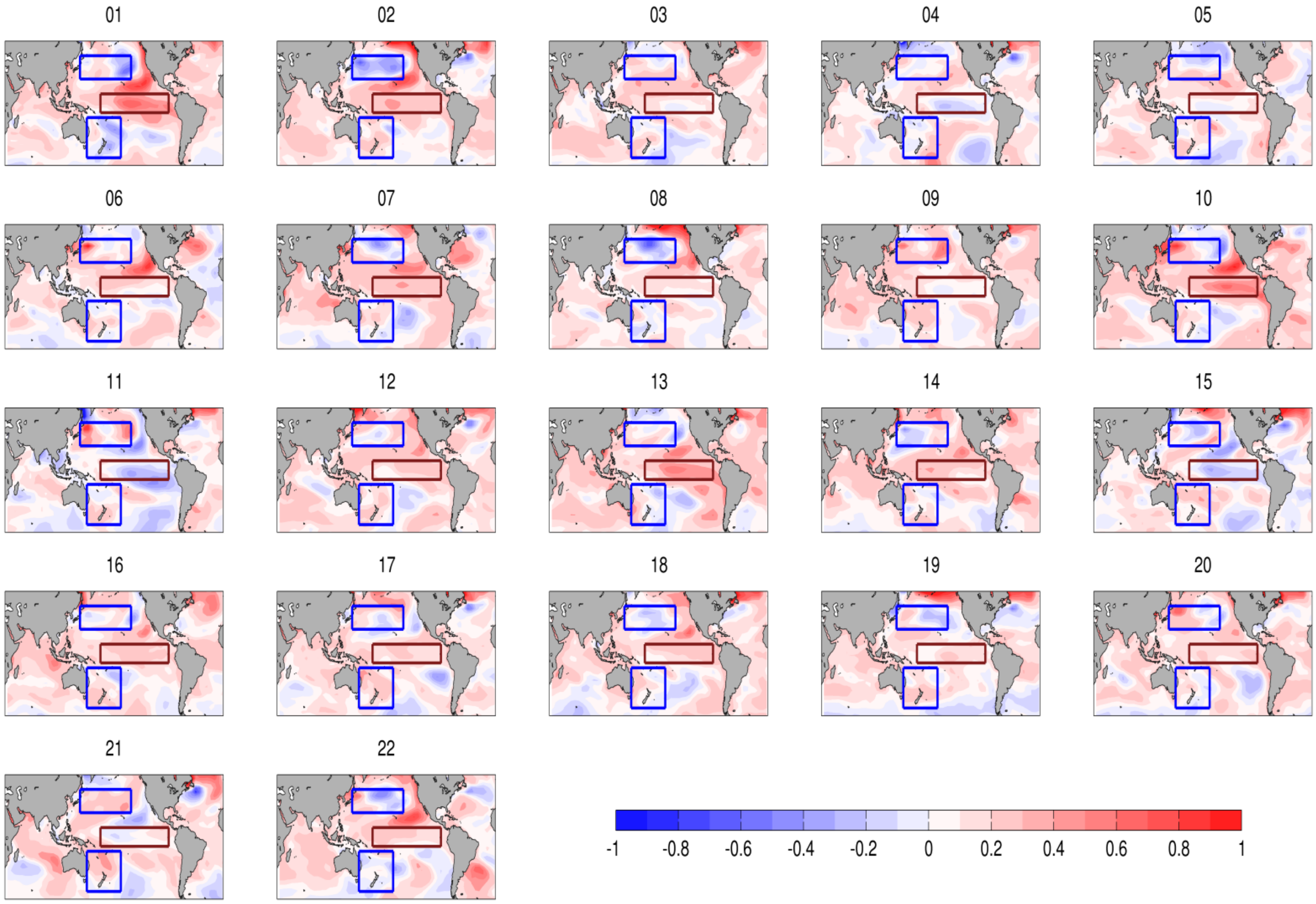

Supplementary Figure 16: 20-year trend in surface air temperature. As in Fig. S13, but for the Mk3l-IPO-Cold.

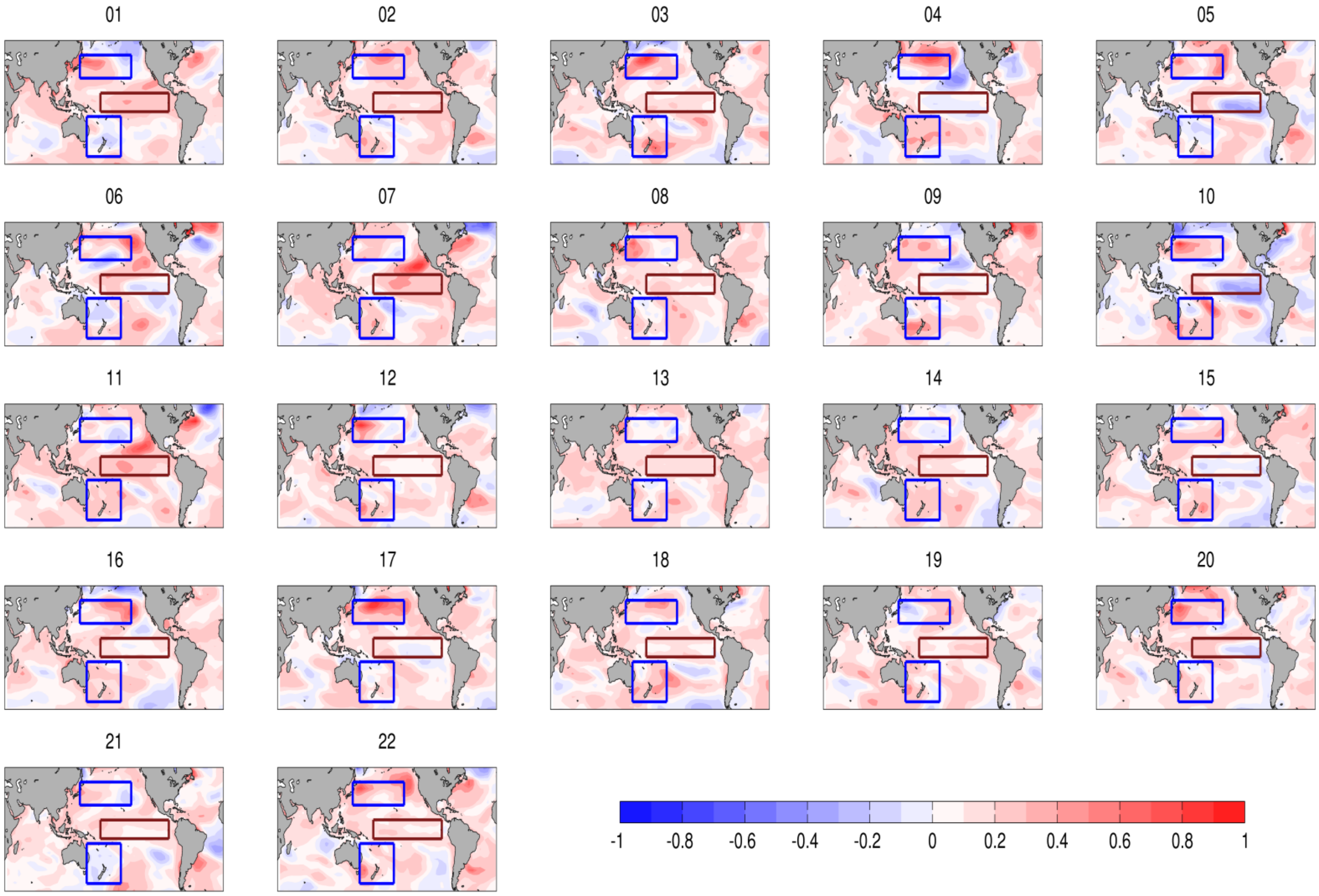

Supplementary Figure 17: 20-year trend in surface air temperature. As in Fig. S13, but for the Mk3l-IPO-Warm.

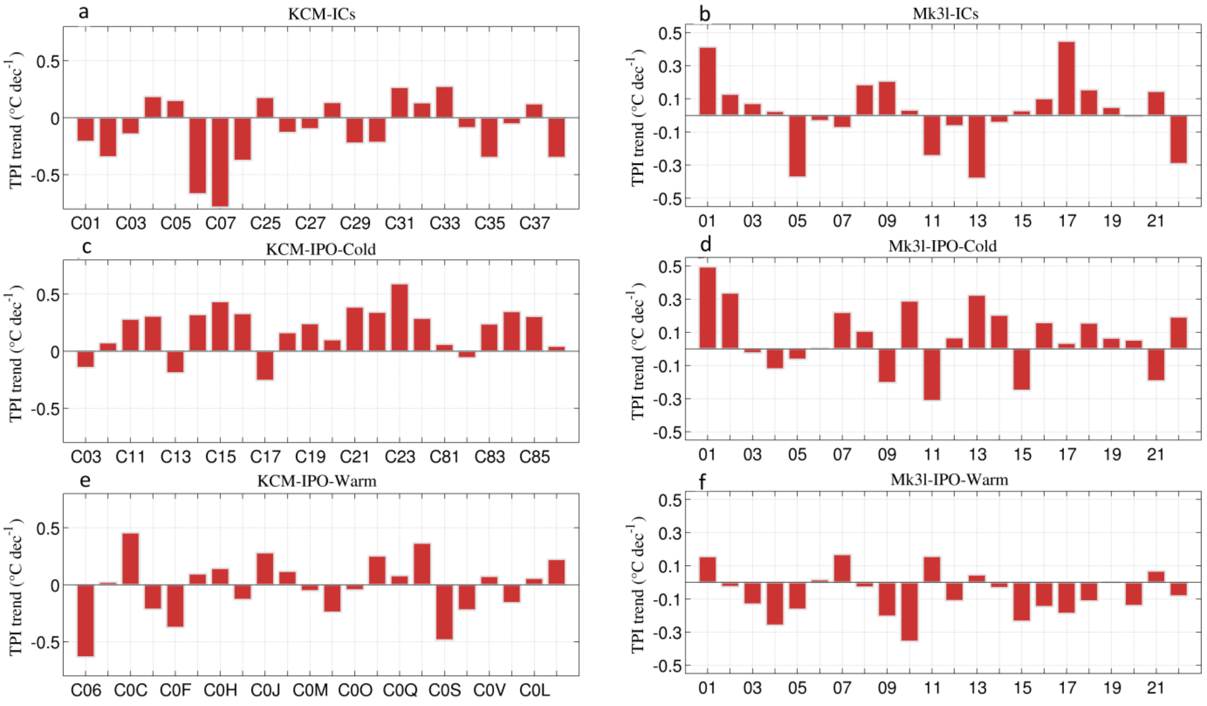

Supplementary Figure 18: Distribution of 20-year trends in the Interdecadal Pacific Oscillation across ensemble members. Shown are the trends in the TPI, defined as the difference between SAT 20-year trends over the central tropical Pacific (10°S-10°N, 170°E-90°W) and that obtained from the averaged over the Southwest (50°S-15°S, 150°E-160°W) and Northwest (50°S-15°S, 150°E-160°W) Pacific in the KCM-ICs (a), the KCM-IPO-Cold (c), the KCM-IPO-Warm (e), the Mk3l-ICs (b), the Mk3l-IPO-Cold (d) and Mk3l-IPO-Warm (f) ensemble of global warming simulations. These regions are shown with rectangular boxes in Fig. S18 and correspond to the TPI regions (Method). The positive (negative) values show the IPO positive-like (IPO negative-like) trends. The x-axes denote the individual ensemble member. Please note that left (a,c,e) and right (b,d,f) panels have different y-axis scales.

Supplementary Table 1: The models, their resolutions and the simulated data sets involved in this study; more technical information of the models is provided in the corresponding references.

Model	Resolution				Ref.$^{\sim}$	Data					
	Ocean		Atmosphere			Ctrl run		Global warming			
	H.$^{+}$	V.$^{++}$	Hor.	Ver.		Year	CO_2 (ppm)	M$^{\sim\sim}$	Scenario	ICs.$^{\$}$	Acronym
**KCM**	~1.3°x1.3°	31	~3.7°x3.7°	19	[3]	1000	348	22	1% CO_2	**	KCM-ICs
								22	1% CO_2	***	KCM-IPO-Cold
								22	1% CO_2	***	KCM-IPO-Warm
**CSIRO-Mk3L**	~2.8°x1.6°	21	~5.6°x3.2°	18	[4]	1100	280	22	1% CO_2	**	Mk3l-ICs
								22	1% CO_2	***	Mk3l-IPO-Cold
								22	1% CO_2	***	Mk3l-IPO-Warm
**CSEM1-CAM5**	~1.0°x1.0°	60	~1.0°x1.0°	30	[5]	1800	280	35	Obs. & RCP8.5	**	CESM-Hind-Proj

$^{+}$*: Horizontal,*$^{++}$*: Vertical,* $^{\sim}$*: Reference,* $^{\sim\sim}$*: number of ensemble member,* $^{\$}$*: Initial condition,* ***: Different Oceanic and Atmospheric ICs,* ****: Fixed Oceanic ICs, perturbed atmospheric ICs*

Supplementary Table 2: The cross correlation between the time series of the globally averaged surface air temperature (GMT), zonal wind stress over the tropical Pacific (see Method section) Niño3.4 index and the Tripole index for the IPO (TPI; see Method section) obtained from the KCM, CESM1-BGC and CSIRO-Mk3l control runs used in this study. Italic numbers in brackets denote the p-value derived from the statistical t-student test. The null hypothesis indicates no correlation between the time series. 1-P-value represents the significance level of rejecting null hypothesis.

**Model**	correlation			
	GMT & wind stress	GMT & TPI	TPI & wind stress	Niño3.4 and wind stress (annual mean)
KCM	0.31 *(0.001)*	0.32 *(0.001)*	0.65 *(0.001)*	0.86 *(0.001)*
CESM1-BGC	0.42 *(0.001)*	0.33 *(0.001)*	0.81 *(0.001)*	0.95 *(0.001)*
CSIRO-Mk3l	0.56 *(0.001)*	0.46 *(0.001)*	0.90 *(0.001)*	0.92 *(0.001)*

Supplementary Table 3: Standard deviation (STD), the maximum (Max), the minimum (Min), the range and the mean of the 20-yr trends in GMT obtained from the KCM-ICs, KCM-IPO-Cold, Mk3l-ICs and CESM-Hind-Proj ensemble of global warming simulations. The trend in the CESM-Hind-Proj is computed for the periods corresponding to 1992-2011 and 2016-2035 and referenced to the hindcast and forecast periods. The trend in the KCM-ICs, KCM-IPO-Cold, Mk3l-ICs are obtained from the first 20-yrs of integration. The range is the difference between the minimum and maximum and the relative uncertainty is the ratio between the range and the mean.

Ensemble name	STD (°C/dec)	Max (°C/dec)	Min (°C/dec)	Range (°C/dec)	Mean (°C/dec)	Relative Uncertainty
CESM-Hind-Proj (hindcast)	0.050	0.374	0.135	0.238	0.256	0.93
CESM-Hind-Proj (forecast)	0.052	0.456	0.213	0.243	0.375	0.65
KCM-ICs	0.062	0.368	0.100	0.268	0.247	1.08
KCM-IPO-Cold	0.042	0.448	0.290	0.158	0.348	0.45
Mk3l-ICs	0.042	0.264	0.088	0.175	0.178	0.98

References in Supplementary Material:

1. Bordbar, M. H. et al. Effects of long-term variability on projections of twenty-first century dynamic sea level. Nature Climate Change, 5(4), 343–347 (2015).
2. Bindoff, N.L. et al. Detection and Attribution of Climate Change: from Global to Regional. In: Climate Change 2013: The Physical Science Basis. Contribution of Working Group I to the Fifth Assessment Report of the Intergovernmental Panel on Climate Change Cambridge University Press, Cambridge, United Kingdom and New York, NY, USA (2013).
3. Park, W. et al. Tropical Pacific Climate and Its Response to Global Warming in the Kiel Climate Model. J. Climate, 22, 71–92 (2009).
4. Phipps, S. J. et al. Paleoclimate data-model comparison and the role of climate forcings over the past 1500 years. J. Clim., 26, 6915–6936 (2013).
5. Kay, J. E. et al. The Community Earth System Model (CESM) Large Ensemble Project: A Community Resource for Studying Climate Change in the Presence of Internal Climate Variability. Bull. Amer. Meteor. Soc., 96, 1333–1349 (2015).
